# A Multipurpose Study of BaZrS_3_ and BaHfS_3_: Absolute Entropies, Thermal Decomposition, and Prediction of Intrinsic and Extrinsic Thermodynamic Stability

**DOI:** 10.1021/acs.jpcc.6c01064

**Published:** 2026-05-08

**Authors:** Alexis Gibson, Andrea Ciccioli, Natalie Parkinson, Riccardo Testa, Corrado Di Conzo, Marco Rossi, Alessandro Latini, Stefano Vecchio Ciprioti, Hasan Arif Yetkin, Phillip J. Dale, Brian Woodfield, Lorenza Romagnoli

**Affiliations:** † Department of Chemistry and Biochemistry, 6756 Brigham Young University, Provo, Utah 84602-2401, United States; ‡ Department of Chemistry, Sapienza University of Rome, Rome 00185, Italy; § Department of Applied Science and Technology (DISAT), Polytechnic of Turin, Turin 10129, Italy; ∥ Department of Basic and Applied Sciences for Engineering (SBAI), Sapienza University of Rome, Rome 00161, Italy; ⊥ Research Centre for Nanotechnology Applied to Engineering (CNIS), Sapienza University of Rome, Rome 00185, Italy; # Department of Physics and Materials Science, University of Luxembourg, Belvaux 4422, Luxembourg

## Abstract

Chalcogenide perovskites have emerged in the past few years as very promising candidates for photovoltaic applications. However, experimental studies on these materials are still relatively scarce and there is a concerning lack of experimentally determined physical properties, which is particularly serious for thermodynamic properties, notwithstanding their importance in the assessment of the suitability of these materials for the proposed applications. In this work, the thermodynamic properties of chalcogenide perovskites BaZrS_3_ and BaHfS_3_ were therefore studied with the aim of estimating the intrinsic stability of the two compounds and the thermodynamic tendency to react under relevant synthesis/real world environments. The measurement of heat capacities from 1.8 to 300 K was performed for the first time and absolute entropies of the two materials were derived therefrom. Furthermore, the thermal decomposition of BaZrS_3_ was investigated by means of Knudsen effusion mass spectrometry up to 1850 K, revealing the release of gaseous sulfur as the only gaseous decomposition product up to about 1600 K. The largely dominant sulfur species were S­(g) and S_2_(g), as expected for a low sulfur-activity phase, with higher oligomers S_3_–S_8_ only observed in the very first steps of heating. Above 1600 K, also BaS­(g) was identified in the vapor phase, with an activity lower than unity, suggesting that pure solid BaS is not formed upon thermal degradation. XRD, SEM, TEM and Raman analyses performed on the residual sample indicated the formation of the Ruddlesden–Popper phase Ba_2_ZrS_4_, confirming previous theoretical predictions. However, no isothermal invariance of the sulfur partial pressure was observed, making it impossible to identify any heterogeneous equilibrium established under the effusion conditions. Finally, by combining the newly determined absolute entropies of the two chalcogenide perovskites with theoretical formation energies available in the literature, the intrinsic thermodynamic stability of BaZrS_3_ and BaHfS_3_ and the thermodynamic degradation behavior under oxygen, water, and water + CO_2_ gaseous atmospheres were predicted. Both BaZrS_3_ and BaHfS_3_ were shown to be stable with respect to the binary sulfides BaS, ZrS_2_, and HfS_2_ at room temperature, with the entropic term causing further stabilization at higher temperatures.

## Graphical abstract

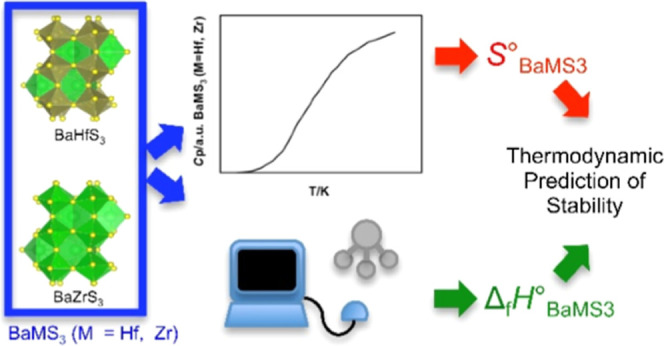

## 1 Introduction

Starting from the work published in 2015 by Sun et al.,^1^ in which chalcogenide perovskites ABCh_3_ (Ch = group VI divalent anion) were proposed for photovoltaic applications, based on their band gaps and optical absorption properties, this class of inorganic semiconductors has gained an ever-increasing attention as a potential alternative to metal halide hybrid perovskites for the top cell in the tandem application.^2^ In fact, while the latter type of materials, whose prototypical compound is methylammonium lead triiodide, has reached, in less than two decades, excellent photovoltaic performances on a laboratory and industrial scale, they also, unfortunately, revealed almost immediately serious drawbacks to their commercialization, mainly due to the lack of thermal and chemical stability.^3^

The potential for optoelectronic and other functional applications of ABO_3_ crystalline materials with perovskite structure had been already recognized previously.^4^ Indeed, oxide perovskites constitute a widely investigated class of materials: their multifarious properties, such as ferroelectricity, ferromagnetism, thermal conductivity and superconductivity, have been known for decades,^5^ and practical applications as catalysts^6,7^ were also reported. In addition, their thermal and chemical stability are desirable characteristics in view of long-term utilization. Regretfully, the band gap values of oxide perovskites, in most cases above 3 eV, are too large to allow efficient solar light harvesting for photovoltaic applications, the optimal value for maximum photo conversion efficiency being around 1.34 eV, according to the Shockley–Queisser limit.^8^

However, the optoelectronic properties of chalcogenide perovskites containing anions other than oxide have received practically no attention until the publication of the aforementioned paper by Sun et al.,^1^ although the earliest reported synthesis and structure determination of BaZrS_3_, BaTiS_3_ and SrTiS_3_ ternary sulfides date back to 1957,^9^ and a few other compounds of this class were prepared and crystallographically characterized subsequently.^10,11^ Among the reasons which hindered the study of these materials, there are the very demanding conditions needed for their preparation, in terms of high temperatures and toxic reagents.^2^ Despite the notable improvements achieved in very recent years in the synthesis methodologies for the attainment of both powders and thin films,^12^ as a matter of fact, most of the research work on this class of materials is carried out by means of first-principles calculations^13–15^ and many of their fundamental properties have never been experimentally determined.

Table 1 summarizes the most important structural and electronic characteristics measured for BaZrS_3_ and BaHfS_3_, two of the most investigated chalcogenide perovskites and the subject of the present study.

**Table 1:** Experimental Structural and Electronic Properties of BaZrS_3_ and BaHfS_3_

material	space group	a/Å	b/Å	c/Å	Unit cell volume/Å^3^	Band gap/eV
BaZrS_3_	*Pnma*	7.0599^a^	9.9813^a^	7.0251^a^	495.0376^a^	1.75–1.94^b^
BaHfS_3_	*Pnma*	7.0020^a^	9.9150^a^	6.9950^a^	485.6267^a^	2.06–2.17^b^

a Data are taken from ref 11.

b The complete set of values can be found in ref 2.

The lack of experimentally determined properties is especially true for thermodynamic properties. While the formation energy from binary precursors of a number of chalcogenide perovskites has been calculated by means of DFT by several groups^14–20^ and is also reported in online repositories, such as the Materials Project^21^ and Open Quantum Materials Database,^22,23^ no experimental formation energy values have been reported so far. Furthermore, in all these studies only perovskite stability with respect to binary precursors is predicted, whereas competing formation or decomposition reactions are not considered, and entropy contributions are usually neglected. An interesting approach has been recently used in the work by Kayastha et al.,^24^ where also the Gibbs energy changes for decomposition of BaZrS_3_ into ZrS_2_ and competing 2D Ruddlesden–Popper phases Ba_*n*+1_Zr_*n*_S_3*n*+1_ (with *n* = 1, 2, 3) were evaluated, by using an ab initio thermodynamic model previously applied to Cu_2_ZnSnS_4_ (CZTS).^25^ Subsequently, by the same approach, this group predicted the Gibbs free energy of formation of BaZrS_3_, both from the elements in the solid state and from binary precursors, and also studied the effect of sulfur vapor pressure on the formation of the perovskite, of different binary reaction intermediates and of competing phases.^26^

The experimental assessment of the thermal stability of chalcogenide perovskites and the measurement of their thermodynamic properties is crucial to test the accuracy of computational models, and also to provide helpful information on viable conditions for thin film deposition, which is essential for real applications. In this regard, we report the determination of thermodynamic functions of two of the most promising chalcogenide perovskites, namely BaZrS_3_ and BaHfS_3_, from low temperature heat capacity measurements, in the present work. On this basis, the intrinsic and extrinsic thermodynamic stability of these materials in different environmental conditions was evaluated. Additionally, thermal decomposition of BaZrS_3_ was studied for the first time by the Knudsen effusion mass spectrometry (KEMS) technique, which allowed the identification of gaseous species released at high temperature and the measurement of their partial pressures.

## 2 Methods

### 2.1 Synthesis of BaZrS_3_ and BaHfS_3_

All the reagents were used as received. Barium sulfide powder (99.9% purity), hafnium powder 325 mesh (99.5% purity excluding 2–4% Zr), sulfur powder (99.98% purity) and toluene (ACS-ISO grade) were purchased from Sigma-Aldrich-Merck. Zirconium powder 60–100 mesh (99.8% purity) was purchased from Chempur. Wheaton 10 mL prescored clear borosilicate glass ampules were purchased from Sigma-Aldrich-Merck. Materials were prepared according to a previously published procedure.^27,28^ BaS, Zr or Hf, and S were ground in a 1:1:3 ratio in an agate mortar for 20 min, then the mixture was transferred to a glass ampule, previously dried in an oven at 150 °C, and evacuated by a rotary pump to 3·10^–1^ mbar, while heating to about 80 °C by a blow dryer. The ampule connected to the vacuum pump was flame-sealed with a butane torch, inserted in an alumina crucible and annealed in a muffle furnace heated at 500 °C for 16 h. Subsequently, the crucible with the ampule was taken out, while still at 500 °C, let cool down to room temperature, then the ampule was opened, the product recovered and ground in an agate mortar, washed with cold water, filtered under suction, washed three times with small amounts of acetone and dried. If excess sulfur is present, the solid is transferred in a beaker and stirred for 1 h in toluene at 85 °C to remove the elemental sulfur, filtered under suction and dried in air. X-ray diffraction patterns (Figure S1a,b) of the so obtained powders (black for BaZrS_3_ and red for BaHfS_3_) confirmed the purity of products.

### 2.2 Powder X-ray Diffraction

Diffraction patterns of the as-synthesized BaZrS_3_ and BaHfS_3_ and of the solid residue left after KEMS experiments on BaZrS_3_ were acquired with a Malvern Panalytical X’Pert Pro MPD diffractometer (Cu Kα radiation, λ = 1.54184 Å), operating in Bragg–Brentano geometry and equipped with an ultrafast X’Celerator RTMS detector. The angular range 2θ = 10–90° was used in all the measurements.

### 2.3 Thermogravimetry-Differential Thermal Analysis

Simultaneous TG-DTA on BaZrS_3_ and BaHfS_3_ were carried out with a Netzsch STA409 PC Luxx thermal analyzer, in flowing Ar atmosphere (100 cm^3^·min^–1^ @STP, purity >99.9995%) with a heating rate of 10 K·min^–1^ from room temperature to 600 °C, using alumina crucibles. Thermograms were recorded for the as-such synthesized samples and after a treatment in high vacuum up to about 330 °C, performed on the samples destined for heat capacity measurements.

### 2.4 Raman Spectroscopy

Raman measurements have been performed at room temperature at various spots on the solid residue obtained after KEMS experiments on BaZrS_3_, using a Renishaw inVia Reflex Raman Microscope configured in backscattering geometry. The powder sample was placed into the openings of a 100-mesh copper grid, which was affixed to double-sided carbon tape, ensuring that the powder sticked to the carbon tape. All Raman spectra were acquired using a 50× objective with two different excitation wavelengths: 633 nm (grating: 2400 L/mm) and 785 nm (grating: 1200 lines/mm). Furthermore, a silicon wafer as a calibration sample was used to calibrate each laser wavelength. In order to exclude any laser-induced damage on the sample, visual inspections were done on each measurement spot before and after each measurement. All obtained Raman spectra were normalized to 1 according to highest intensity.

### 2.5 Heat Capacity Measurements

Heat capacity measurements were performed on a Quantum Design Physical Property Measurement System (PPMS) in zero magnetic field from 1.8 to 300 K. The samples were prepared according to a method devised for measuring the heat capacities of insulating powders.^29,30^ Samples were enclosed in copper cups (0.025 mm thick, 99.999% purity from Alfa Aesar) with two small copper coils of the same purity inserted in each cup to ensure uniform heating throughout the samples. After being pressed into a pellet, the sample was attached to the sample holder using a small amount of Apiezon N grease and placed in the PPMS. An addenda measurement was performed before the measurement to account for the heat capacity of the sample holder and the grease. This method has an estimated accuracy of ±2% below 10 K and ±1% from 10 to 300 K. The sample and copper masses used are listed in Table 2.

**Table 2:** Details of the PPMS Calorimetric Measurements Including Pressures (*p*), Sample Mass (*M*_s_), and Copper Mass (*M*_Cu_)­^a^

	*p*/mPa	*M*_s_/mg	*M*_Cu_/mg
BaHfS_3_	1.2	9.18	22.97
BaZrS_3_	1.2	10.64	21.07

a The estimated standard uncertainties in the masses *M*_s,Cu_ and pressures *p* are *u*(*M*_s,Cu_) = 0.06 mg and *u*(*p*) = 0.1 mPa.

### 2.6 Knudsen Effusion Mass Spectrometry

KEMS experiments were carried out on BaZrS_3_, synthesized according to the procedure detailed previously, with a single focusing 90° magnetic sector mass spectrometer, originally manufactured by Patco, equipped with a Knudsen molecular source, as described in.^31^ The sample was placed in a tungsten effusion cell, provided with a lid with a small orifice (0.95 mm diameter), enclosed in an external tantalum crucible. A spiral-shaped tungsten heating element surrounding the crucible was used. The crucible and the heating coil were in turn surrounded by several thermal shields made of tantalum foil. Ultrahigh vacuum is produced in the apparatus (typical residual pressure in the ion source region: 5·10^–7^ mbar) by a turbomolecular and three ion pumps. The temperature was measured by a W5% Re–W26% Re (type C) thermocouple, inserted in a small hole drilled in the bottom of the external crucible. Ionization efficiency curves were measured for all the detected ions and appearance energies (i.e., the lowest value of the electron energy necessary to produce an ion) were determined with the vanishing current method, by correcting the observed values based on the appearance energy of the ion Au^+^, taken as reference.^32^ Ba-containing ions also showed some thermal contribution (i.e., they were produced by heating and not only by electron impact), which was assessed by switching off the electron-emitting filament and subtracted to total intensity for partial pressure calculations (see eq 1 below). The energy of the ionizing electrons used was adjusted based on the maximum ionization efficiency and was between 8 and 20 eV. A movable shutter is placed between the molecular source and the spectrometer, allowing one to distinguish ions from the Knudsen cell from background species. Ion intensities were taken as the difference between total intensities and those measured with the shutter shifted so as to exclude the ions from the cell. Partial pressures *P*_*i*_ of species *i* in the gas phase were obtained by applying eq 1

1 $$P_{i}=\frac{k_{instr}}{\sigma_{i}}\sum_{k}\frac{I_{k}^{+}T\sqrt{M_{k}}}{a_{k}}$$

In this equation, *I*_k_^+^are ion currents of ions k (which can be either molecular ions or fragments) produced from neutral species *i* upon electron impact, *M*_k_ is their molecular mass, *a*_k_ the isotope abundance of the monitored isotope of k, *T* is the absolute temperature, σ_*i*_ is the ionization cross section of *i*, and *k*_instr_ is an instrumental constant, whose value is given by $\frac{P_{ref}a_{ref}\sigma_{ref}}{I_{ref}^{+}\sqrt{M_{ref}}T_{ref}}$ , derived from calibration experiments with a reference substance of known vapor pressure *P*_ref_. In the experiments described in this work, calibration was carried out by vaporizing Au (99.99% purity), supplied by Engelhard, and Ag (purity 99.999%), supplied by Alfa Aesar. Sulfur activity in the perovskite was estimated by the equation

2 $$a_{S}=\sqrt[{m-n}]{\frac{p_{S_{m}}}{p_{S_{n}}}}$$

where *S*_m_ and *S*_n_ are two oligomers formed in the gas phase.

As discussed in Section 3.2, a KEMS experiment was also carried out, for the sake of comparison, on pure BaS, under the same experimental conditions used for BaZrS_3_.

### 2.7 Transmission and Scanning Electron Microscopy

TEM analysis was performed on pristine BaZrS_3_ and the residue after KEMS experiments. Each sample was prepared by dispersing 3 mg of powder in 8 mL of hexane, sonicating for 40 min, and depositing a few drops of this dispersion onto a TEM grid. Measurements were performed with an F200 JEOL Multipurpose transmission electron microscope equipped with a GATAN Rio16 CMOS camera and a JEOL EDX spectrometer.^33^ Electron diffraction patterns were indexed using CrysTBox software with the DiffractGUI tool.^34^ SEM images of both powdered samples were acquired using a Thermo Fisher Phenom ProX equipped with an EDX spectrometer for compositional analysis.

### 2.8 Thermodynamic Calculations

Simulations aimed at predicting the thermodynamic stability of BaZrS_3_ under various reactive atmospheres (see Section 3.3) were carried out using the POLY-3 module (a software for equilibrium calculations based on a Gibbs energy minimization algorithm) of the Thermo-Calc package, version L.^35^ To this end, a user database was assembled by extracting the thermodynamic data retrieved in the SGTE Substance database SSUB94 ^36^ and by adding the Gibbs energy function *G*(*T*)–*H*_SER_(298) for the BaZrS_3_ phase as given by the following expressions: −1102175 + 11.748·*T* – 0.3338·*T* ^2^ – 0.00025653·*T* ^3^ + 8.138·10^–7^·*T* ^4^ up to 298.15 K and −1071922.6–166.93·*T* – 0.069449·*T* ^2^ from 298.15 to 500 K. The first equation was obtained by a simplified fit of the data measured in the present work, whereas the high temperature equation was estimated by using for the enthalpy of formation from the elements at 298.15 K the value −1077 kJ·mol^–1^ (see Section 3.3 and eq 13 therein) and by approximating the heat content and entropy functions at *T* > 298.15 K by the additivity rule C_p_(BaZrS_3_) = C_p_(BaS) + C_p_(ZrS_2_) corrected by a term accounting for the difference with our experimental values at 298.15 K. The other solid phases included in the database are the following: BaCO_3_, BaC_2_, Ba­(OH)_2_, BaH_2_, BaO, BaO_2_, BaZrO_3_, BaSO_4_, BaS, Ba, ZrC, C (graphite), C (diamond), ZrO_2_, ZrS_2_, Zr_2_S_3_, S, Zr.

## 3 Results and Discussion

### 3.1 Heat Capacities and Thermodynamic Functions of BaZrS_3_ and BaHfS_3_

Heat capacity measurements were carried out on samples that had been previously heated up to 600 K in high vacuum for about 4 h to remove any possible residual excess sulfur. TG-DTA measurements were performed under inert atmosphere before and after the treatment. The thermograms, reported as Figure S2a,b, indicate that the treatment successfully removed the volatile sample impurities; X-ray diffraction patterns confirmed that no phase change occurred during heating.

The measured heat capacities for each sample are listed in Tables S1 and S2. Figure 1 shows the measured heat capacities for each sample with their theoretical fits and Figure S3 shows the data’s deviations from the fits based on Table 3. The heat capacity data were fitted using theoretical functions in two distinct temperature regions (*T* < 10 K and *T* > 40 K). An orthogonal polynomial was used to smoothly transition between the two regions. Fitting parameters are given in Table 3, and thermodynamic functions at smoothed temperatures based on the fits are given in Tables 4 and 5.

**Figure 1:**
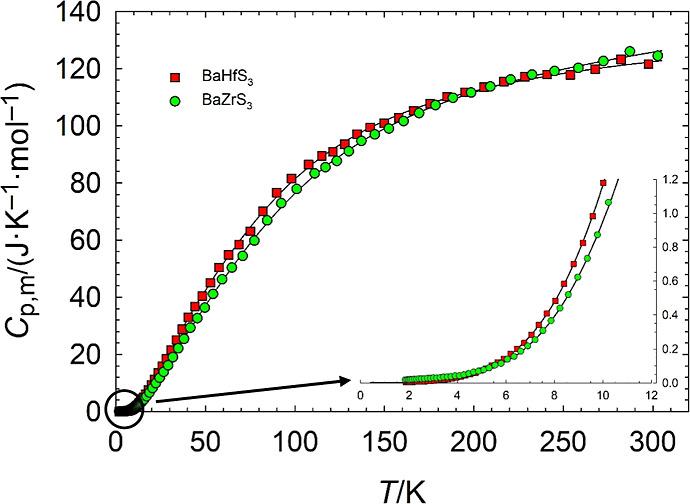
Measured molar heat capacity as a function of temperature for BaHfS_3_ and BaZrS_3._ The black curve placed behind the heat capacity is a plot of the theoretical fits in Table 3. The inset shows the points at *T* < 10 K. The point removed due to error in the heat capacity of grease at high temperatures is included in the plot of the raw data for BaHfS_3_, but is not included as part of the data used in the calculation of the theoretical fits.

**Table 3:** Parameters for Low*T* (<10 K), mid *T* (5 K < *T* < 65 K), and High*T* (*T* > 40 K) Fits of Heat Capacity Data (in J·K^–1^·mol^–1^) for BaHfS_3_ and BaZrS_3_ ^a^

	Parameter	BaHfS_3_	BaZrS_3_
Low *T* Fits	*B*_–3_/J·K^2^·mol^–1^	–0.07618	–0.39307
	*B*_–2_/J·K·mol^–1^	0.04514	0.25762
	*B*_3_/J·K^–4^·mol^–1^	2.8704·10^–4^	4.5928·10^–4^
	*B*_5_/J·K^–6^·mol^–1^	1.2574·10^–5^	2.5424·10^–6^
	*B*_7_/J·K^–8^·mol^–1^	–3.9500·10^–8^	2.8626·10^–8^
	% RMS	1.86	2.70
	Range/K	1.87–8.29	1.85–6.75
Mid *T* Fits	*A*_0_/J·K^–1^·mol^–1^	–1.7753	–1.4612
	*A*_1_/J·K^–2^·mol^–1^	1.1430	0.95464
	*A*_2_/J·K^–3^·mol^–1^	–0.28108	–0.23555
	*A*_3_/J·K^–4^·mol^–1^	0.033108	0.027779
	*A*_4_/J·K^–5^·mol^–1^	–1.8195·10^–3^	–1.5348·10^–3^
	*A*_5_/J·K^–6^·mol^–1^	5.6048·10^–5^	4.7571·10^–5^
	*A*_6_/J·K^–7^·mol^–1^	–9.8874·10^–7^	–8.4268·10^–7^
	*A*_7_/J·K^–8^·mol^–1^	9.3047·10^–9^	7.9411·10^–9^
	*A*_8_/J·K^–9^·mol^–1^	–3.6153·10^–11^	–3.0814·10^–11^
	% RMS	0.52	0.91
	Range/K	8.29–53.48	6.75–45.08
High *T* Fits	*B*/J·K^–2^·mol^–1^	0.026567	0.056394
	*m*/mol	2.3399	2.2448
	Θ_D_/K	155.77	178.38
	*n*/mol	2.5259	2.4296
	Θ_E_/K	332.62	359.31
	% RMS	0.95	0.91
	Range/K	53.48–297.56	45.08–302.75

a One Point was Excluded in the High-Temperature Fits of BaHfS_3_.

**Table 4:** Standard Thermodynamic Functions of BaHfS_3_ ^b^

*T*/K	*C*_p,m_/J·K^·^mol^–1^	Δ_0K_^*T*^ *S*_m_°/J·K^·^mol^–1^	Δ_0K_^*T*^ *H*_m_°/kJ·mol^–1^	Φm°^a^/J·K^·^mol^–1^
0	0	0	0	0
2	4.457·10^–3^	8.452·10^–4^	1.281·10^–6^	2.047·10^–4^
3	0.01291	3.182·10^–3^	7.308·10^–6^	7.462·10^–4^
4	0.03223	8.606·10^–3^	2.663·10^–5^	1.949·10^–3^
5	0.07329	0.01938	7.567·10^–5^	4.245·10^–3^
6	0.14962	0.03864	1.825·10^–4^	8.229·10^–3^
7	0.27796	0.07044	3.904·10^–4^	0.01467
8	0.47672	0.11956	7.605·10^–4^	0.02450
9	0.76957	0.19155	1.375·10^–3^	0.03882
10	1.1656	0.29222	2.334·10^–3^	0.05887
15	4.5514	1.3331	0.01574	0.28391
20	9.4587	3.2922	0.05038	0.77340
25	14.930	5.9850	0.11122	1.5361
30	20.627	9.2077	0.20003	2.5399
35	26.476	12.826	0.31776	3.7468
40	32.236	16.740	0.46466	5.1237
45	37.546	20.849	0.63935	6.6414
50	42.393	25.058	0.83930	8.2721
60	51.836	33.636	1.3112	11.782
70	60.490	42.286	1.8735	15.522
80	68.351	50.886	2.5184	19.406
90	75.339	59.349	3.2376	23.376
100	81.459	67.611	4.0223	27.388
110	86.773	75.630	4.8641	31.411
120	91.370	83.382	5.7553	35.421
130	95.348	90.856	6.6894	39.399
140	98.798	98.052	7.6605	43.334
150	101.80	104.97	8.6639	47.214
160	104.43	111.63	9.6953	51.033
170	106.74	118.03	10.751	54.787
180	108.79	124.19	11.829	58.473
190	110.61	130.12	12.926	62.089
200	112.24	135.84	14.041	65.634
210	113.70	141.35	15.171	69.109
220	115.03	146.67	16.314	72.514
230	116.24	151.81	17.471	75.851
240	117.35	156.78	18.639	79.120
250	118.37	161.59	19.818	82.323
260	119.31	166.25	21.006	85.462
270	120.19	170.77	22.204	88.539
273.15	120.45	172.17	22.583	89.495
280	121.00	175.16	23.409	91.554
290	121.77	179.42	24.623	94.511
298.15	122.36	182.80	25.618	96.878
300	122.49	183.56	25.845	97.411

a $\Phi_{m}^{{}^{\circ}}=\Delta_{0K}^{T}S_{m}^{{}^{\circ}}-\frac{\Delta_{0K}^{T}H_{m}^{{}^{\circ}}}{T}$

b All calculated thermodynamic values have an estimated standard uncertainty of 0.02 X below 10 K and 0.01 X at and above 10 K, where X represents the thermodynamic property. *C*_*p*,m_, Δ_0K_^*T*^ *S*_m_°, Δ_0K_^*T*^ *H*_m_°, and Φ_m_° are the heat capacity, standard entropy, standard enthalpy, and Gibbs function, respectively. Nuclear contributions were excluded when calculating thermodynamic functions. Values are reported with extended significant digits to preserve the original measurement precision and facilitate future data fitting and modeling.

**Table 5:** Standard Thermodynamic Functions of BaZrS_3_ ^b^

*T*/K	*C*_p,m_/J·K^·^mol^–1^	Δ_0K_^*T*^ *S*_m_°/J·K^·^mol^–1^	Δ_0K_^*T*^ *H*_m_°/kJ·mol^–1^	Φ_m_°^a^/J·K^·^mol^–1^
0	0	0	0	0
2	0.01903	1.242·10^–3^	1.865·10^–6^	3.090·10^–4^
3	0.02715	4.266·10^–3^	9.633·10^–6^	1.055·10^–3^
4	0.04243	0.01039	3.136·10^–5^	2.545·10^–3^
5	0.07475	0.02105	7.978·10^–5^	5.089·10^–3^
6	0.13232	0.03817	1.746·10^–4^	9.069·10^–3^
7	0.22936	0.06460	3.474·10^–4^	0.01498
8	0.39126	0.10500	6.517·10^–4^	0.02354
9	0.63017	0.16408	1.156·10^–3^	0.03567
10	0.95227	0.24641	1.940·10^–3^	0.05242
15	3.7191	1.0965	0.01289	0.23733
20	7.7506	2.6994	0.04123	0.63792
25	12.306	4.9120	0.09123	1.2628
30	17.164	7.5806	0.16478	2.0878
35	22.291	10.609	0.26334	3.0850
40	27.465	13.925	0.38778	4.2301
45	32.329	17.445	0.53745	5.5014
50	37.102	21.099	0.71108	6.8774
60	46.223	28.678	1.1282	9.8754
70	54.797	36.455	1.6337	13.115
80	62.737	44.298	2.2220	16.523
90	69.951	52.111	2.8860	20.044
100	76.408	59.822	3.6185	23.637
110	82.132	67.378	4.4118	27.271
120	87.183	74.746	5.2589	30.922
130	91.636	81.904	6.1534	34.570
140	95.568	88.842	7.0898	38.200
150	99.051	95.556	8.0633	41.801
160	102.15	102.05	9.0696	45.365
170	104.93	108.33	10.105	48.885
180	107.42	114.40	11.167	52.357
190	109.68	120.27	12.253	55.777
200	111.74	125.95	13.360	59.145
210	113.62	131.44	14.487	62.457
220	115.36	136.77	15.632	65.715
230	116.96	141.93	16.794	68.917
240	118.46	146.94	17.971	72.064
250	119.86	151.81	19.163	75.157
260	121.17	156.53	20.368	78.196
270	122.42	161.13	21.586	81.183
273.15	122.79	162.55	21.972	82.113
280	123.59	165.60	22.816	84.119
290	124.71	169.96	24.058	87.004
298.15	125.59	173.43	25.078	89.319
300	125.78	174.21	25.310	89.840

a $\Phi_{m}^{{}^{\circ}}=\Delta_{0K}^{T}S_{m}^{{}^{\circ}}-\frac{\Delta_{0K}^{T}H_{m}^{{}^{\circ}}}{T}$

b All calculated thermodynamic values have an estimated standard uncertainty of 0.02 X below 10 K and 0.01 X at and above 10 K, where X represents the thermodynamic property. *C*_p,m_, Δ_0 K_^*T*^ *S*_m_°, Δ_0 K_^*T*^ *H*_m_°, and Φ_m_° are the heat capacity, standard entropy, standard enthalpy, and Gibbs function, respectively. Nuclear contributions were excluded when calculating thermodynamic functions. Values are reported with extended significant digits to preserve the original measurement precision and facilitate future data fitting and modeling.

Variations in the high temperature data points are believed to be caused by uncertainty in the heat capacity of the Apiezon N grease at high temperatures.^29^ To correct for this problem, one of the high temperature data points for BaHfS_3_ was removed from the data when calculating the theoretical fits for this region.

The heat capacity of the BaHfS_3_ and BaZrS_3_ samples are represented below 10 K by

3 $$C_{p,m}=B_{-3}T^{-3}+{B_{-2}T^{-2}+B}_{3}T^{3}+B_{5}T^{5}+B_{7}T^{7}$$

where *B*_–3_, *B*_–2_, *B*_3_, *B*_5_, and *B*_7_ are constants obtained from fitting the data. The *B*_3_ *T* ^3^ *,B*_5_ *T* ^5^, and *B*_7_ *T* ^7^ terms represent the lattice contribution to the heat capacity and account for any anharmonicity in lattice phonons.^37^ The *B*_–3_ and *B*_–2_ terms are taken as the first two terms in the series expansion of the low-temperature limit of a Schottky anomaly, commonly found in compounds with a nuclear hyperfine field.^37,38^ The *B*_–3_ and *B*_–2_ terms were excluded when calculating the thermodynamic functions to focus on chemical contributions.

The middle temperature regions were fitted to eighth order orthogonal polynomials, which do not have a theoretical basis but are used to provide a smooth overlap between the low and high temperature functions^39^

4 $$C_{p,m}=\sum_{i=0}^{8}A_{i}T^{i}$$

Heat capacity data in the high temperature region (*T* > 40 K) were fitted with a combination of Debye and Einstein functions, which represent the contribution of lattice vibrations at higher temperatures

5 $$C_{p,m}=BT+m\cdot D\left(\Theta_{D}/T\right)+n\cdot E\left(\theta_{E}/T\right)$$

where *D*(Θ_D_/*T*) is a Debye function and *E*(Θ_E_/*T*) is an Einstein function, and *m* and *n* represent the number of Debye or Einstein oscillators with characteristic temperatures of Θ_D_ and Θ_E_ respectively.^39^ The *B* term is used to correct for the difference in isochoric and isobaric heat capacities.^40^

### 3.2 Thermal Decomposition of BaZrS_3_ Studied by Knudsen Effusion Mass Spectrometry

Vaporization of BaZrS_3_ by Knudsen effusion mass spectrometry was undertaken with the main aim to ascertain the nature of the vapor phase released from this compound at high temperature under close-to-equilibrium conditions, thus getting information on the thermodynamic prerequisites for possible vapor deposition processes of thin films.

Three consecutive experiments were carried out on the same BaZrS_3_ sample. In the first experiment, the sample was mildly heated, up to *T* = 462 K, and only ions formed from sulfur oligomers up to S_8_^+^ (with prevalence of S_2_^+^ and S^+^, the latter being, at least in part, a fragment of S_2_(g)) were detected in the mass spectrum of the perovskite. The values of sulfur activities (Figure S4), being in the range 0.1–0.8 in the first stage of the measurement and rapidly dropping to ∼10^–17^ after 2 h of heating (with disappearing of all oligomers higher than S_2_), indicate the initial presence of minimal amounts of excess sulfur impurities from the synthesis, under the detection limit of PXRD.

The subsequent experiments have been performed at higher temperatures, up to 1294 K for the second run and up to 1852 K for the third. By increasing temperature, a change in the composition of the vapor was observed. Sulfur was found exclusively in the form of monomer and dimer, S­(g) and S_2_(g), and also Ba-containing molecules started to be released from temperatures around 1600 K. As an instance, the spectrum of the gas phase produced from BaZrS_3_ at 1829 K is shown in Figure 2.

**Figure 2:**
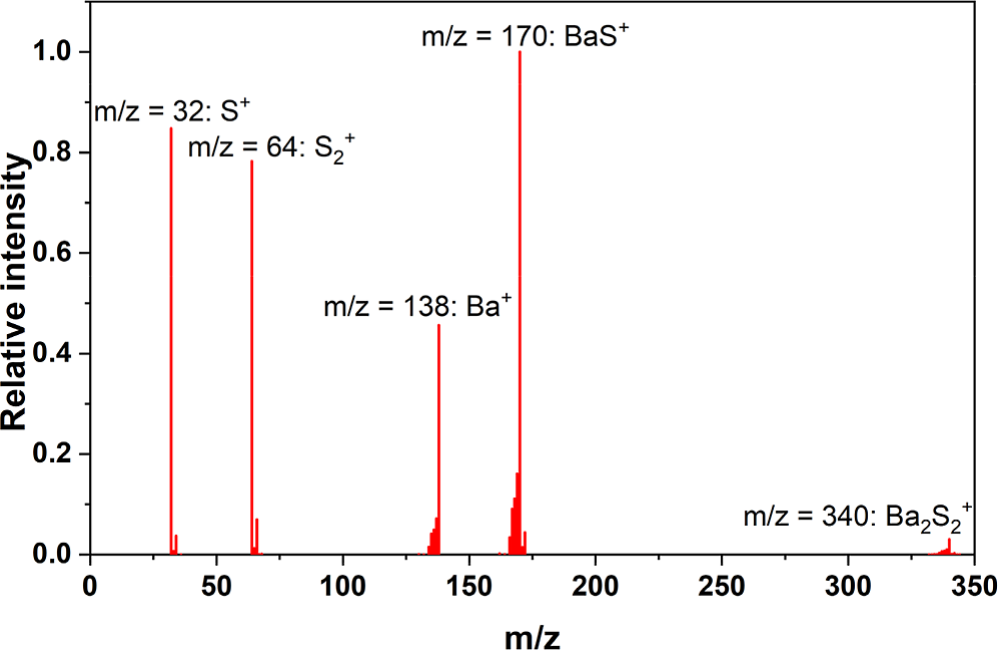
Mass spectrum of the vapor phase above BaZrS_3_ at *T* = 1829 K.

Figure 2 shows the presence of the ion species S^+^, S_2_^+^, Ba^+^, BaS^+^ and Ba_2_S_2_^+^ (the latter with a very low intensity), while no Zr-containing species were detected in the gas phase. This clearly indicates the (expected) occurrence of incongruent vaporization, confirming the impossibility to deposit a thin film of BaZrS_3_ from the bulk powder, at least under near-equilibrium conditions.

Partial pressures of neutral species in the vapor were estimated by means of eq 1. Note that measured ion intensities of Ba-containing species showed a contribution from thermal ionization which was subtracted to obtain the electron impact signal *I*_k_^+^ to be used in eq 1. Ionization cross sections were taken from ref 41. The so-evaluated partial pressures of gaseous species released by BaZrS_3_ at the various temperatures for the three experiments are reported in Tables S3–S5, in chronological order. It is evident from these values that the composition of the gas phase changes continuously with time for a given temperature, thus indicating that a monovariant heterogeneous equilibrium inside the cell is not attained, as also suggested by the already mentioned variation of sulfur activity as a function of time and temperature (Figure S4).

In order to verify if BaS(s) was formed upon decomposition of the BaZrS_3_, its activity was estimated from the ratio of BaS partial pressures measured via BaZrS_3_ vaporization and those obtained in a separate experiment on pure BaS (see Figure S5), $a_{BaS}=\frac{p_{BaS}^{BaZrS_{3}}}{p_{BaS}^{BaS}}$ . The comparison between partial pressures of BaS in the BaZrS_3_ and those of pure BaS is shown in Figure 3. As can be inferred from the comparison of partial pressures, the values of BaS activity in the BaZrS_3_ (ranging from 0.10 to 0.12) point to the absence of pure BaS(s) in the solid phase formed by thermal decomposition of BaZrS_3_.

**Figure 3:**
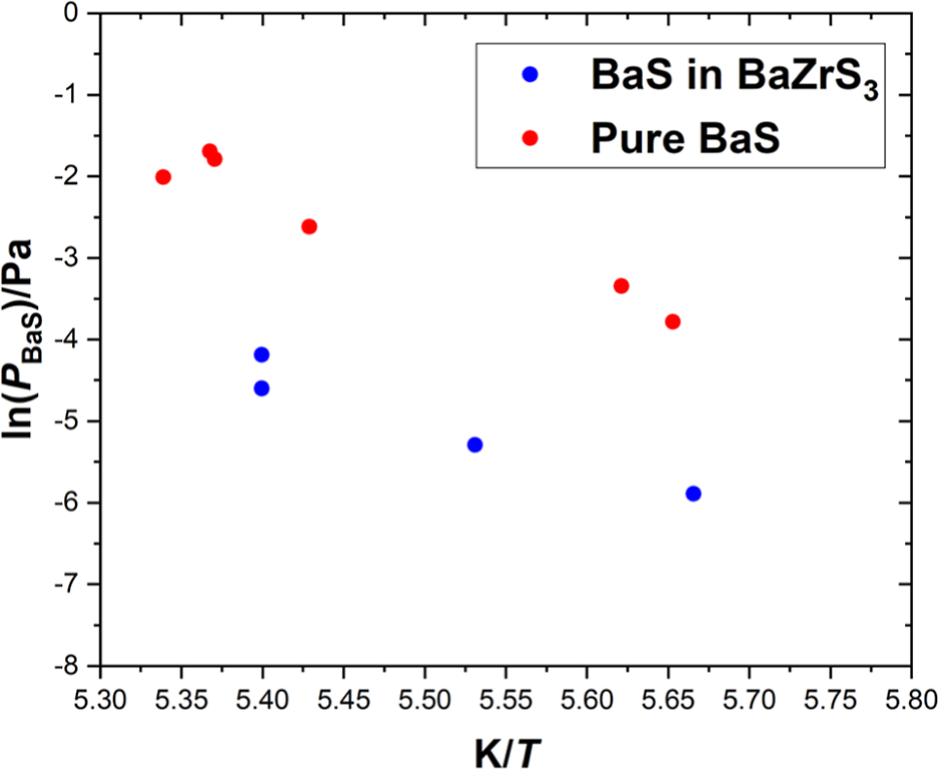
BaS partial pressure *vs* reciprocal temperature for BaZrS_3_ and for pure BaS.

In order to attempt the identification of the decomposition process­(es) occurring during heating and to determine whether there is a chemical interaction between the sample and the crucible material, the residual sample of KEMS experiments was subject to PXRD, SEM, TEM and Raman analyses. PXRD patterns were acquired after each KEMS run, whereas SEM and TEM and Raman spectra were performed on the final residue only. The PXRD patterns in Figure S6 display, besides the peaks of residual BaZrS_3_, a number of new peaks whose intensities gradually increase from the first to the third residue. However, the only Ba–Zr–S phase that could be unambiguously assigned was the 2D Ruddlesden–Popper Ba_2_ZrS_4_ phase. Stoichiometric considerations could suggest the simultaneous formation of a Zr–S binary phase. Nevertheless, the remaining peaks in the residue are not clearly attributable to any known zirconium sulfide pattern. This made it impossible to identify any heterogeneous equilibrium established in the effusion cell and to derive the corresponding thermodynamic properties. The presence of tungsten-containing phases that could form from possible interaction with the crucible material was ruled out. Note that the absence of pure BaS in the PXRD pattern seems to confirm the low activity of BaS in the solid phase, consistently with the BaS­(g) partial pressure data discussed above. Based on Raman spectra of the residue sample (see here below) the formation of ZrO_2_ in nanocrystalline form (not detected in PXRD patterns) is suggested.

TEM and SEM analysis of KEMS residue compared with those of pristine sample revealed a higher degree of crystallinity in the former, due to high temperature crystallization, evidenced by a more ordered disposition of lattice planes in TEM images of the residue and a more regular shape of the grains in SEM images of the same sample, as can be seen in Figure 4a–d.

**Figure 4:**
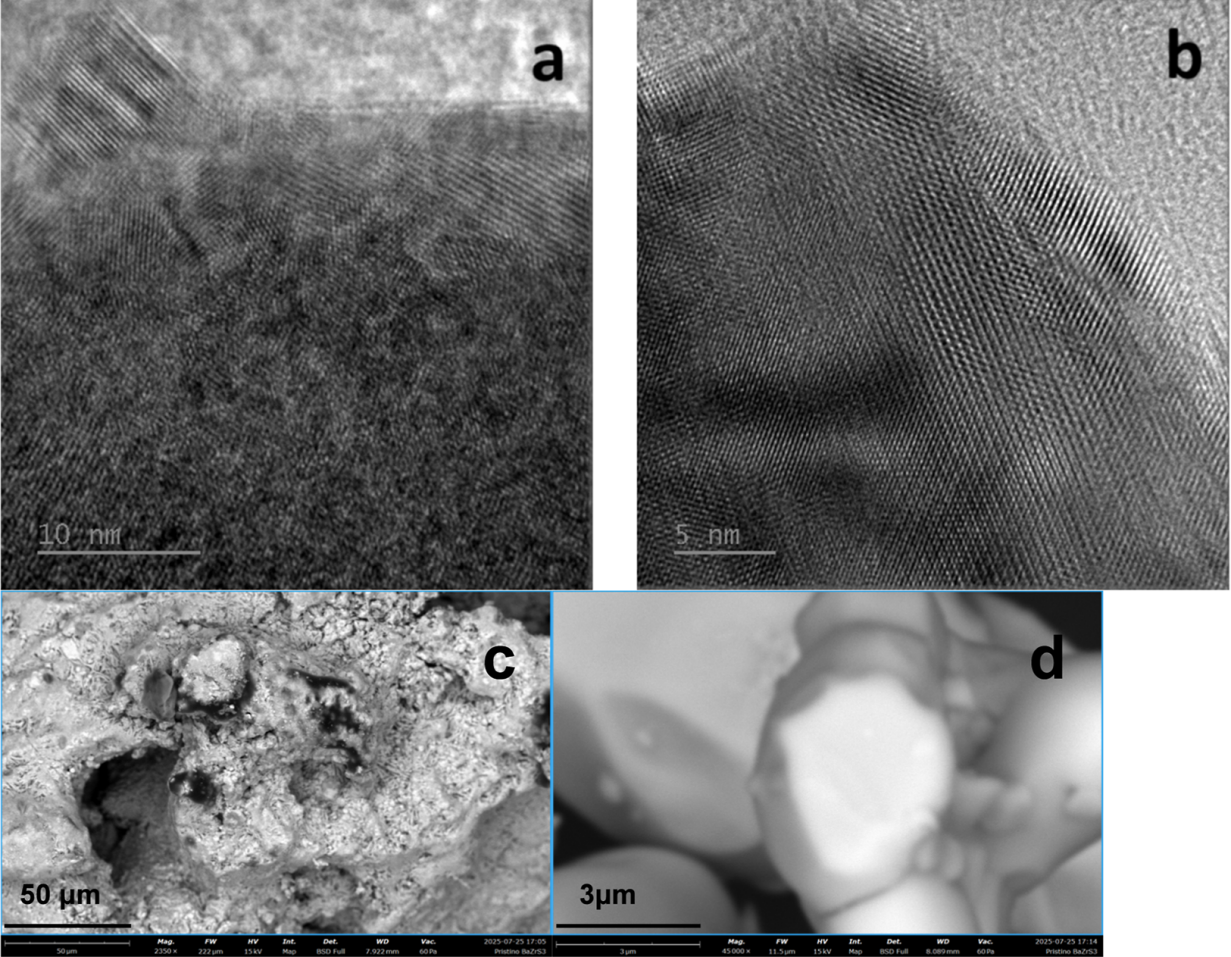
TEM images of (a) pristine BaZrS_3_ and (b) residue from KEMS experiments, and SEM images of (c) pristine and (d) KEMS residue.

Electron diffraction obtained by TEM from polycrystalline pristine BaZrS_3_ confirmed phase purity, in agreement with SEM–EDX analysis (Table S6). Conversely, analysis of the KEMS residue identified only the residual BaZrS_3_ and 2D Ruddlesden–Popper Ba_2_ZrS_4_ among the possible binary and ternary phases.

The presence of Ba_2_ZrS_4_ is confirmed by its electron-diffraction pattern (Figure S7), which matches the reference pattern.^42^ This finding is also consistent with the PXRD data of the residue and is in reasonable agreement with the SEM–EDX analysis (Table S7). The sulfur abundance determined by EDX (46% at.) could be a slight underestimation or could be affected by other unidentified sulfur-poor phases.

Raman spectra of the residue, performed with excitation wavelength of 633 and 785 nm (Figure 5a,b), revealed the presence of a few phases produced after KEMS experiments. In particular, none of the spectra, performed on various spots of the sample as indicated by numbers 1–3 in the figures, resemble that of BaZrS_3_.^28^ ZrO_2_, possibly formed as a nanocrystalline phase (and therefore difficult to see through XRD) by reaction promoted at high temperature by the residual oxygen partial pressure in the vacuum environment of the KEMS instrument, was identified in at least one spot with the use of 633 nm laser. Notably, the combination of spectra of spots 1 and 2 performed with 633 nm laser roughly gives the spectrum of spot 3, supporting the presence of Ba_2_ZrS_4_ and ZrO_2_ phases, the former identified by comparison with the spectrum reported by Ishii et al.^43^ Similarly, Raman spectra performed with 785 nm laser do not match the reference spectrum of BaZrS_3_ ^28^ nor that of Ba_3_Zr_2_S_7_ reported by Ishii et al.,^43^ while confirming once again the presence of Ba_2_ZrS_4_, in agreement with the previously discussed analysis, in the spot 2. The existence of Ba_2_ZrS_4_, possibly together with an unidentified phase, is also suggested by the spectra, very similar to each other, of spots 1 and 3 performed using 785 nm laser.

**Figure 5:**
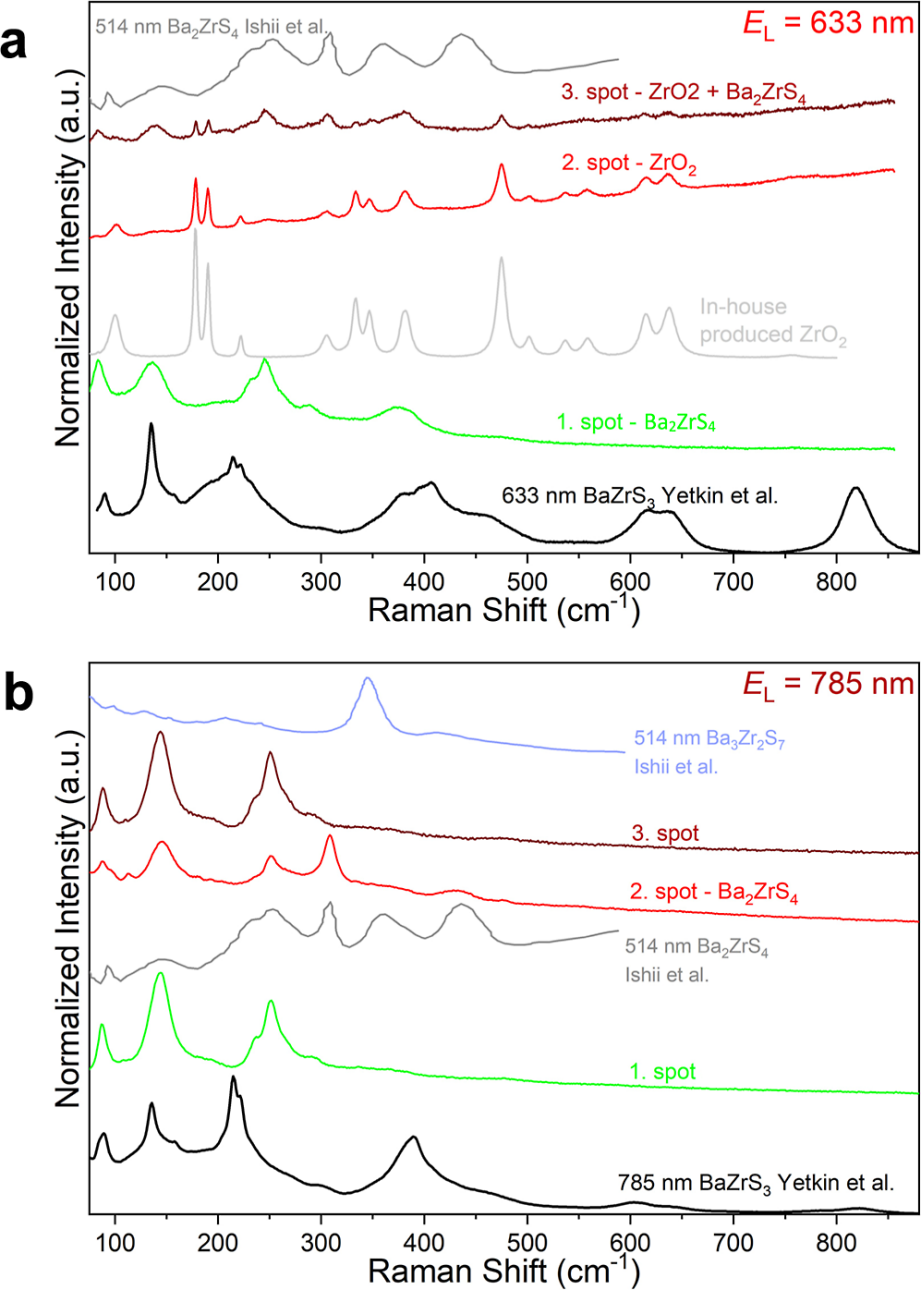
Raman spectra performed on various spots (indicated by numbers 1–3) of the solid residue obtained after KEMS experiments at (a) 633 nm and (b) 785 nm excitation wavelength. Reference spectra of BaZrS_3_ from ref 28 and of Ruddlesden–Popper phases from ref 43 are shown for comparison.

Finally, we used the measured partial pressures to determine the enthalpy change of the isomolecular gas phase equilibrium

6 $$BaS\left(g\right)+S\left(g\right)⇄Ba\left(g\right)+S_{2}\left(g\right)$$

and, from there, the enthalpy of dissociation (bond enthalpy) at 0 K of BaS­(g). Note that this reaction has the advantage of being independent of the instrumental constant in eq 1, *k*_instr_, and therefore is not affected by possible uncertainties in determination of its value.

The Δ_*r*_ *H*_0K_° of reaction (6) was obtained by applying the so-called third-law method of analysis of vapor pressure data^44^

7 $$\Delta_{r}H_{0K}^{{}^{\circ}}=-RT\ln K_{eq}+T\left(\Delta \Phi_{T}^{{}^{\circ}}\right)$$

where Φ_T_° is defined as

8 $$\Phi_{T}^{{}^{\circ}}=-\frac{G_{T}^{{}^{\circ}}-H_{0K}^{{}^{\circ}}}{T}$$

The free energy functions of the various species were taken from the IVTANTHERMO thermochemical database.^45^ The Δ_*r*_ *H*_0K_° of reaction (6) obtained for different temperatures are shown in Table 6.

**Table 6:** Standard Reaction Enthalpy at 0 K of (6) Obtained by Third-Law Treatment of KEMS Data

*T*/K	Δ_*r*_ *H*_0K_°/kJ·mol^–1^
1808	–26.73
1852	–27.81
1852	–27.03

From these values, a mean Δ_*r*_ *H*_0K_° = (−27.2 ± 0.6) kJ·mol^–1^ was obtained, which in turn yielded a dissociation enthalpy of BaS­(g) of (393.4 ± 20.6) kJ·mol^–1^. This value is in good agreement with the only previously reported determination,^46^ Δ_*r*_ *H*_0K_° = (396.2 ± 18.8) kJ·mol^–1^.

### 3.3 Intrinsic and Extrinsic Thermodynamic Stability of BaZrS_3_ and BaHfS_3_

The absolute entropies derived from measured heat capacities of BaZrS_3_ and BaHfS_3_ enable to estimate the Gibbs energy of formation of the two compounds, as well as to perform some thermodynamic predictions on their stability in different conditions and environments.

To the best of our knowledge, no experimental value of the enthalpy of formation from elements (Δ_*f*_ *H*°) of BaZrS_3_ and BaHfS_3_ is available in the literature. Our attempts to estimate these quantities from high-temperature decomposition processes were unsuccessful because no well-defined heterogeneous equilibrium was established. In the past few years, however, a number of computational studies have reported the energy of formation of both perovskites from their binary precursors

9 $$BaS\left(s\right)+{MS}_{2}\left(s\right)\to {BaMS}_{3}\left(s\right)\left(with\;M=Hf,Zr\right)$$

A summary of these theoretical values is given in Table 7. The mean values are −33.4 kJ·mol^–1^ for BaZrS_3_ and −33.9 kJ·mol^–1^ for BaHfS_3_, respectively. To convert these values to the corresponding enthalpy changes at 0 K, the zero point energy and the small *P*Δ*V* term should, in principle, be considered. However, for the purpose of estimating thermodynamic stability, on first approximation these contributions can be neglected. Conversion of the enthalpy difference from 0 to 298 K would require the heat contents of all precursors. Because the heat contents of ZrS_2_ and HfS_2_ are undetermined, this contribution to Δ_*r*_ *H*_298K_° of reactions (eq 9) was also neglected. This approximation is the rough equivalent of assuming additivity of heat capacity values at low temperature (i.e., the Kopp-Neumann rule).

**Table 7:** Energy of Formation (in kJ·mol^–1^) from Binary Sulfide Precursors (eq 9) for BaZrS_3_ and BaHfS_3_ from Computational Works and Online Repositories^a^

BaZrS_3_	BaHfS_3_
–27.9;^14^ −38.5;^15^ −21.3;^16^ −44.0;^18^ −32.7;^19^ −38.5;^20^ −30.6;^21^ −33.8 ^22^	–40.3;^14^ −28.9;^17^ −42.0;^18^ −28.0;^21^ −30.1 ^22^

a References are indicated as superscript next to the values.

As for the entropy change at 298 K of reactions in eq 9, it was evaluated by using the absolute entropies of BaHfS_3_ and BaZrS_3_ in Tables 4 and 5, respectively, the value 78.45 J·K^–1^·mol^–1^ for BaS^45^ and the estimated values of 78.2 and 83.7 J·K^–1^·mol^–1^ for ZrS_2_ ^47^ and HfS_2_,^48^ respectively. The absolute entropies of the two chalcogenide perovskite phases resulted thus to be higher than the sum of the absolute entropies of the binary sulfide precursors BaS and ZrS_2_ or HfS_2_, at least within the limits of accuracy of the latter, which are only available as empirical estimates. The resulting entropy changes for reactions in eq 9 are 16.8 J·K^–1^·mol^–1^ for BaZrS_3_ and 20.7 J·K^–1^·mol^–1^ for BaHfS_3_. The increases in entropy imply that the thermodynamic stability of both perovskites relative to binary sulfides increases with temperature. This trend is in agreement with first-principles lattice dynamics calculations.^26^

The above-described procedure leads us to propose the following estimates for the change in Gibbs energy of reaction (eq 9)­

$$\Delta_{r}G^{{}^{\circ}}\left(9\right)({BaZrS}_{3})=\left(-33.4-16.8{\cdot 10}^{-3}T/K\right){kJ\cdot mol}^{-1}$$ 10

$$\Delta_{r}G^{{}^{\circ}}\left(9\right)({BaHfS}_{3})=\left(-33.9-20.7{\cdot 10}^{-3}T/K\right){kJ\cdot mol}^{-1}$$ 11

The Δ_*r*_ *G*° at 298 K calculated from eqs 10 and 11 are, respectively, −38.5 kJ·mol^–1^ for BaZrS_3_ and −40.1 kJ·mol^–1^ for BaHfS_3_, indicating that both compounds are thermodynamically stable with respect to binary precursors. The only literature value available for comparison is the computational result of −50 kJ·mol^–1^ by Kayastha et al.^26^ for BaZrS_3_, which would indicate a slightly higher stability. Outside of possible inaccuracies in the computational results, uncertainties in eqs 10 and 11 may arise from the estimated entropies of the binary phases. It should be also noted that the Δ_*r*_ *H*° and Δ_*r*_ *S*° values used in eqs 10 and 11 are those at 298 K, with no temperature dependence included, due to the lack of heat capacity data for the perovskite phases at higher temperature.

From eqs 10 and 11 we determine the corresponding expressions for the formation reactions from the elements

$$Ba\left(s\right)+M\left(s\right)+3S\left(s\right)\to {BaMS}_{3}(s)\;\left(with\;M=Hf,Zr\right)$$ 12

using literature enthalpies of formation for the binary sulfides, Δ_*f*_ *H*°(BaS) = −470 kJ·mol^–1^,^45^ Δ_*f*_ *H*°(ZrS_2_) = −573 kJ·mol^–1^,^47^ and Δ_*f*_ *H*°(HfS_2_) = −575 kJ·mol^–1^.^48^ The formation entropies were derived by combining the absolute entropies of perovskites measured in this work with absolute entropies of elements at 298 K. The resulting expressions are the following

$$\Delta_{r}G^{{}^{\circ}}\left(12\right){(BaZrS}_{3})=\left(-1077+24.3\cdot 10^{-3}T/K\right){kJ\cdot mol}^{-1}$$ 13

$$\Delta_{r}G^{{}^{\circ}}\left(12\right)({BaHfS}_{3})=\left(-1079+19.3{\cdot 10}^{-3}T/K\right){kJ\cdot mol}^{-1}$$ 14

These expressions are valid near 298 K within the uncertainties of the enthalpic and entropic terms.

The corresponding formation enthalpy values are −1077 kJ·mol^–1^ for BaZrS_3_ and −1079 kJ·mol^–1^ for BaHfS_3_. For BaZrS_3_, this value compares well with the theoretical value of −1052 kJ·mol^–1^ reported in ref 26. A similar level of agreement is found for the Gibbs energy of formation at 298 K (−1070 kJ·mol^–1^ in this work and −1037 kJ·mol^–1^ in ref 26). Unlike the formation from binary sulfides, the formation of both perovskites from the elements becomes less favorable with increasing temperature, although Δ_*f*_ *G*° remains strongly negative over several hundred kelvins.

Using the resulting Δ_*f*_ *G*° values, we evaluated the extrinsic thermodynamic stability of both compounds by considering a number of potential decomposition reactions in the presence of oxygen, water, and water + CO_2_. Again, all the thermodynamic quantities for the species involved in these reactions were taken from IVTANTHERMO database.^45^ Reactions considered in this evaluation of stability and the corresponding Δ_*r*_ *G*° at 298 K are listed in Table 8. Note that we did not consider BaO as a possible product of oxidation in reactions (1) to (5), due to the low stability of this oxide compared to ZrO_2_ and HfO_2_. This assumption was also validated by the equilibrium calculations reported below.

**Table 8:** Gibbs Energy (in kJ·mol^–1^) and ln *K*_p_ at *T* = 298 K of Possible Decomposition Reactions of BaZrS_3_ and BaHfS_3_

	reaction	Δ_*r*_ *G*° 298 K (M = Zr)	ln (*K*_p_ 298 K) (M = Zr)	Δ_*r*_ *G*° 298 K (M = Hf)	ln (*K*_p_ 298 K) (M = Hf)
1	BaMS_3_(s) + 3O_2_(g) ⇄ BaS(s) + MO_2_(s) + 2SO_2_(g)	–1035	417.9	–1040	419.5
2	BaMS_3_(s) + 7/2O_2_(g) ⇄ BaSO_4_(s) + MO_2_(s) + S_2_O(g)	–1415	571.1	–1419	572.7
3	BaMS_3_(s) + 4O_2_(g) ⇄ BaSO_4_(s) + MO_2_(s) + 2SO(g)	–1371	553.5	–1376	555.2
4	BaMS_3_(s) + 5O_2_(g) ⇄ BaSO_4_(s) + MO_2_(s) + 2SO_2_(g)	–1929	778.6	–1933	780.2
5	BaMS_3_(s) + 6O_2_(g) ⇄ BaSO_4_(s) + MO_2_(s) + 2SO_3_(g)	–2071	835.9	–2075	837.5
6	BaMS_3_(s) + 4H_2_O(l) ⇄ Ba(OH)_2_(s) + MO_2_(s) + 3H_2_S(g)	24.40	–9.847	20.36	–8.219
7	BaMS_3_(s) + 3H_2_O(l) + CO_2_(g) ⇄ BaCO_3_(s) + MO_2_(s) + 3H_2_S(g)	–99.90	40.32	–103.9	41.95

As can be seen from the values in Table 8, all the reactions with oxygen at 298 K are extremely favored from a thermodynamic point of view. Reaction with water and CO_2_ is also favored at the reference temperature, although Δ_*r*_ *G*° is much less negative, compared with reactions with oxygen. The very negative Δ_*r*_ *G*° of reactions of BaZrS_3_ and BaHfS_3_ with oxygen may appear surprising, considering that experiments show long-term stability of both compounds at ambient atmosphere.^49^ The observed stability is most likely due to a very high kinetic barrier that hinders decomposition under typical storage conditions. Among the selected decomposition processes, only reaction with water is not favored thermodynamically at 298 K according to reaction 6 in Table 8. In a recent paper, BaZrS_3_ was found to decompose in aqueous environments due to sulfur oxidation.^50^ However, additional thermodynamic driving force under these conditions could be due to the formation of ionic species, not considered in reaction 6. Interestingly, theoretical calculations in ref 50 indicate that sulfur loss from perovskite can hardly be energetically explained by the formation of sulfur vacancies, and an important role of O_2_ adsorption is suggested instead. Indeed, we calculate for the Δ_*r*_ *G*°of reaction BaZrS_3_(s) + 2H_2_O­(l) + 2O_2_(g) ⇄ ZrO_2_(s) + BaSO_4_(s) + 2H_2_S­(g) a value of −1292 kJ·mol^–1^, which indicate a clear thermodynamic enhancement of sulfur oxidation in the presence of oxygen.

As regards the first reaction reported in Table 8 for BaZrS_3_, an interesting comparison can be made with the work by Bystrický et al.:^51^ based on thermogravimetric analysis, these authors propose this process as the first of a two-step mechanism of decomposition of the perovskite at high temperature, and they give Δ_*r*_ *H*° = −1090 kJ·mol^–1^, in very good agreement with our estimate of −1084 kJ·mol^–1^.

Finally, from the Δ_*r*_ *S*°of these decomposition reactions, it was also possible to evaluate their temperature dependence, as shown in Figure 6a,b.

**Figure 6:**
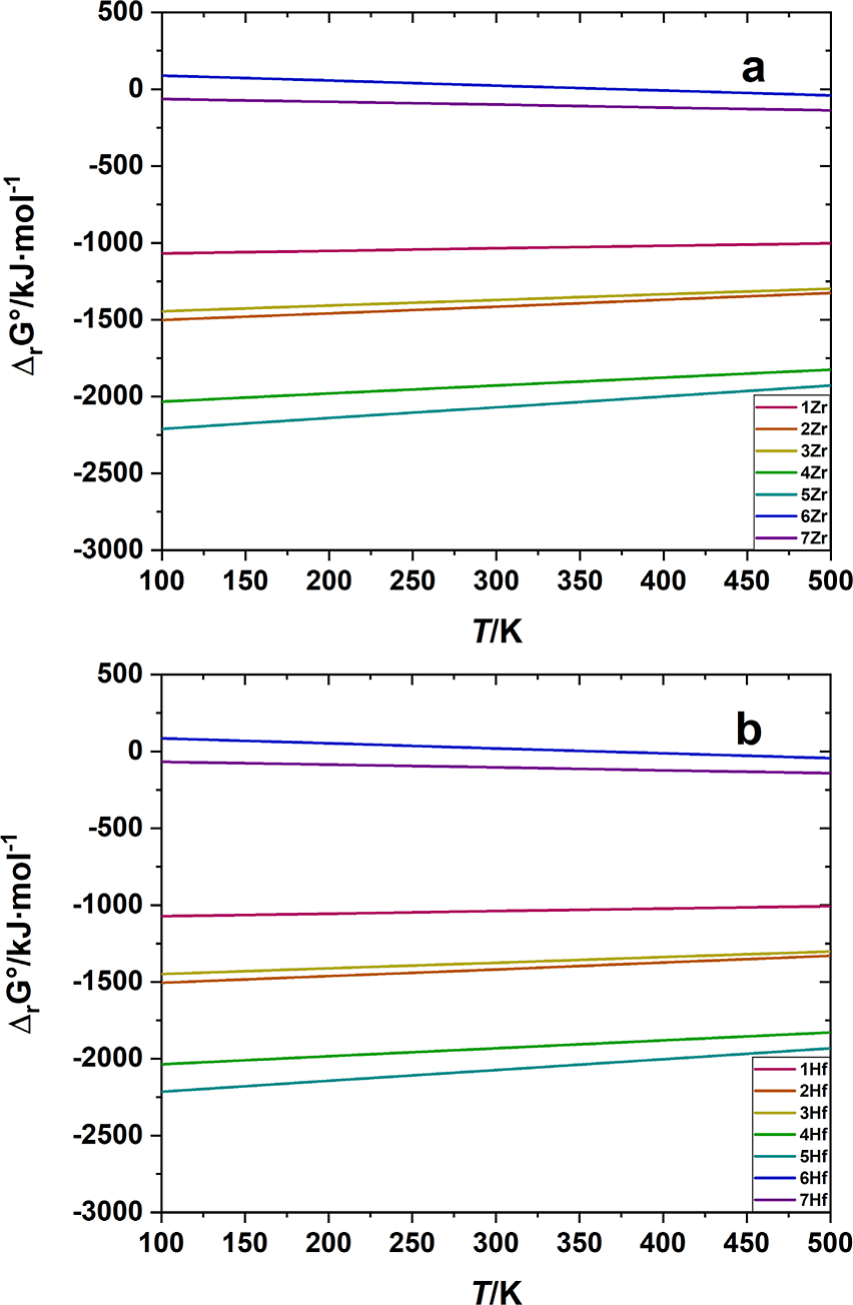
Temperature dependence of Δ_*r*_ *G*° of reactions in Table 8 for (a) BaZrS_3_ and (b) BaHfS_3_. The symbols 1Zr, 2Zr etc. refer to the reaction number in the table.

As shown in Figure 6, while all the reactions with oxygen are more thermodynamically favored (more negative Δ_*r*_ *G*°) at low temperature, reactions with water become favored with increasing temperature. Finally, the decomposition process with water and CO_2_, having a slightly negative Δ_*r*_ *G*° at low temperature, becomes more and more favored with increasing temperature.

The standard Gibbs energy changes discussed above provide a rather clear idea of the thermodynamic tendency toward degradation. However, the most direct method to investigate the thermodynamically driven degradation paths is based on the prediction of the final (equilibrium) conditions, as provided by the nature and composition of the phases resulting from decomposition, attained from the material under given initial conditions. To this end, we performed a number of equilibrium calculations by using the Gibbs energy minimization module POLY-3 of the Thermo-Calc software package (see Section 2.8 for details) to gain more insight into the degradation behavior of BaZrS_3_.

The starting conditions were selected to test the material stability (i) under atmospheric and reduced oxygen pressure; (ii) at room temperature, at a plausible operation temperature under intense heating from the Sun (350 K), and at 500 K as a harsher thermal condition; (iii) under saturated water pressure and 400 ppm for CO_2_. The selected conditions are reported in Table 9 together with the set of solid phases formed at equilibrium and the composition of the produced gas phase. Only the most abundant species are reported for the latter. As can be easily seen, barium sulfate, zirconium oxide, and barium sulfide are the solid phases whose formation is thermodynamically favored under most conditions, whereas the formation of zirconium sulfide seems not to occur. However, it should be remembered that the phase identification and the thermodynamic description of Zr–S phases is far from being complete and the only phases included in the database are ZrS_2_ and Zr_2_S_3_. Even more important, no ternary Ba–Zr–S phase, except BaZrS_3_, is included in the database, due to the complete lack of thermodynamic information on 2D phases. This limitation seems particularly severe in view of the results presented in Section 3.2. On the other hand, the formation of Ba­(OH)_2_ under saturated water environment is not thermodynamically allowed. As expected, in the presence of CO_2_ (at the typical atmospheric concentration), barium carbonate is preferentially formed. With regard to the equilibrated vapor phase, hydrogen sulfide is the main degradation product, both under H_2_O­(g) and under H_2_O­(g) + CO_2_(g) atmospheres. Interestingly, the largely prevailing species in the gas phase produced under an oxygen atmosphere are the homonuclear sulfur oligomers, with no significant presence of sulfur oxides in the explored temperature range.

**Table 9:** Thermodynamic Degradation Pattern of BaZrS_3_ under Selected Reactive Atmospheres at 298, 350, and 500 K^b^

298 K						350 K						500 K					
Initial conditions			Equilibrium conditions			Initial conditions			Equilibrium conditions			Initial conditions			Equilibrium conditions		
*p*O_2_	pH_2_O	*p*CO_2_	Cond. phases^a^	Gas (x_i_)	*p* tot	*p*O_2_	pH_2_O	*p*CO_2_	Cond. phases^a^	Gas (x_i_)	*p* tot	*p*O_2_	pH_2_O	*p*CO_2_	Cond. phases^a^	Gas (x_i_)	*p* tot
21000	-	-	BaSO_4_, BaZrS_3_, ZrO_2_, S	S_8_ (0.77), S_6_ (0.21), S_7_ (0.018)	3.58·10^–4^	21000	-	-	BaSO_4_, BaZrS_3_, ZrO_2_,S	S_8_ (0.76), S_6_ (0.21), S_7_ (0.033)	0.152	21000	-	-	BaSO_4_, BaZrS_3_, ZrO_2_,S (liquid)	S_8_ (0.63), S_6_ (0.27), S_7_ (0.1)	938
100	-	-	BaSO_4_, BaZrS_3_, ZrO_2_, S	S_8_ (0.77), S_6_ (0.21), S_7_ (0.018)	3.58·10^–4^	100	-	-	BaSO_4_, BaZrS_3_, ZrO_2_,S	S_8_ (0.76), S_6_ (0.21), S_7_ (0.033)	0.152	100	-	-	BaSO_4_, BaS, BaZrS_3_, ZrO_2_	S_6_ (0.51), S_8_ (0.35), S_7_ (0.011)	13.3
-	3132	-	BaSO_4_, BaZrS_3_, ZrO_2_	H_2_S (≈1)	3122	-	47373	-	BaS, BaZrS_3_, ZrO_2_	H_2_S (0.9996),H_2_O (3.4· 10^–4^), H_2_ (2.4·10^–5^)	47434	-	2645000	-	BaS, BaZrS_3_, ZrO_2_	H_2_S (0.992), H_2_O (5.7·10^–3^), H_2_S_2_ (1.7·10^–3^)	2640000
-	100	-	BaSO_4_, BaZrS_3_, ZrO_2_	H_2_S (≈1)	100	-	100	-	BaSO_4_, BaZrS_3_, ZrO_2_	H_2_S (0.998), S_8_ (1.1·10^–3^), S_6_ (3.2 ·10^–4^)	100	-	100	-	BaZrS_3_, ZrO_2_	H_2_S (0.999), H_2_ (5.9·10^–4^), H_2_S_2_ (4.0·10^–4^)	99.8
-	3132	40	BaCO_3_, BaS, BaZrS_3_, ZrO_2_	H_2_S (0.99), CH_4_ (5.2·10^–3^), H_2_O (5.6·10^–5^)	3118	-	47373	40	BaCO_3_, BaS, BaZrS_3_, ZrO_2_	H_2_S (0.999), H_2_O (3.4· 10^–4^), H_2_S_2_ (5.5·10^–5^)	47434	-	2645000	40	BaS, BaZrS_3_, ZrO_2_	H_2_S (0.992), H_2_O (5.7·10^–3^), H_2_S_2_ (2.1·10^–3^)	2640000

a Note that the formation of ternary Ba–Zr–S phases other than BaZrS_3_ cannot occur in the simulation, because no 2D ternary phases are included in the database owing to the lack of any thermodynamic data.

b All Pressures are Given in Pa.

## 4 Conclusions

Absolute entropies and other thermodynamic properties of chalcogenide perovskites BaZrS_3_ and BaHfS_3_ have been determined for the first time by measuring isobaric heat capacity from 1.8 to 300 K. Additionally, thermal degradation of the BaZrS_3_ perovskite was investigated in the temperature range 462–1852 K under effusion conditions, and the gas species released from decomposition were identified by mass spectrometry. The vapor phase was found to contain only sulfur, as S­(g) and S_2_(g) species, up to about 1600 K, while, above this temperature, the species Ba­(g), BaS­(g) and Ba_2_S_2_(g) were also observed in the mass spectrum. The activities of sulfur and BaS were estimated as a function of temperature and the dissociation energy of BaS­(g) was determined. XRD diffraction patterns, TEM, SEM–EDX and Raman analyses performed on the residual samples of KEMS experiments revealed the formation of the Ruddlesden–Popper phase Ba_2_ZrS_4_, confirming previous theoretical predictions. However, no isothermal invariance of pressures was observed, making it impossible to perform a thermodynamic analysis of partial pressure data.

By combining the absolute entropies determined in this work with energies of formation retrieved from previous computational works, it was possible to estimate the Gibbs energy of formation of the two perovskites both from binary sulfide precursors and from the elements and to evaluate their stability in various conditions. Both BaZrS_3_ and BaHfS_3_ were shown to be stable with respect to the binary sulfides BaS, ZrS_2_ and HfS_2_ at room temperature, with the entropic term causing further stabilization at higher temperatures. Reaction with oxygen is extremely favored at room temperature from a thermodynamic point of view, yielding barium sulfate and zirconium oxide, although oxidation is not observed experimentally, most probably due to kinetic hindrance. The same products are formed under water + oxygen atmosphere, whereas barium carbonate is formed under water + carbon dioxide atmosphere.

## Supplementary Material



## Notes

Powder diffraction patterns of the as-prepared BaZrS_3_ and BaHfS_3_ with reference patterns (Figure S1); thermogravimetric curves of BaZrS_3_ and BaHfS_3_ before and after heat treatment in high vacuum (Figure S2); molar heat capacities measured for BaHfS_3_ and BaZrS_3_ (Tables S1 and S2); deviation plots of the heat capacity results from the theoretical fits for BaHfS_3_ and BaZrS_3_ (Figure S3); sulfur activity as a function of time and temperature in KEMS experiments on BaZrS_3_ (Figure S4); mass spectrum of the vapor phase above BaS (Figure S5); partial pressures of gaseous species released by BaZrS_3_ in KEMS experiments (Tables S3–S5); XRD pattern of the as-prepared BaZrS_3_ and of solid residues of the same sample after KEMS experiments (Figure S6); experimental Ba_2_ZrS_4_ electron diffraction pattern overlapped to the reference one (Figure S7); results of SEM–EDX analysis on the as-prepared BaZrS_3_ and on the solid residue after KEMS experiments (Tables S6–S7) (PDF)
