# Supplementary material for: A Multipurpose Study of BaZrS3 and BaHfS3: Absolute Entropies, Thermal Decomposition, and Prediction of Intrinsic and Extrinsic Thermodynamic Stability
Source: J Phys Chem C Nanomater Interfaces. 2026 May 8;130(20):7153–66. doi: 10.1021/acs.jpcc.6c01064 (PMC13205216; doi:10.1021/acs.jpcc.6c01064)
Supplement: Supplementary file 1 [file jp6c01064_si_001.pdf]

Supporting information

A Multipurpose Study of $BaZrS_3$ and $BaHfS_3$: Absolute Entropies, Thermal Decomposition, and Prediction of Intrinsic and Extrinsic Thermodynamic Stability

Alexis Gibson,[a] Andrea Ciccioli[b,*], Natalie Parkinson[a], Riccardo Testa,[b] Corrado Di Conzo,[c,d] Marco Rossi,[d,e] Alessandro Latini,[b] Stefano Vecchio Ciprioti,[d] Hasan Arif Yetkin,[f] Phillip J. Dale,[f] Brian Woodfield,[a,*] Lorenza Romagnoli[b,*]

[a] *Department of Chemistry and Biochemistry, Brigham Young University, Provo, Utah, 84602-2401, United States*
[b] *Department of Chemistry, Sapienza University of Rome, Rome, 00185, Italy*
[c] *Department of Applied Science and Technology (DISAT), Polytechnic of Turin, Turin, 10129, Italy*
[d] *Department of Basic and Applied Sciences for Engineering (SBAI), Sapienza University of Rome, Rome, 00161, Italy*
[e] *Research Centre for Nanotechnology Applied to Engineering (CNIS), Sapienza University of Rome, Rome, 00185, Italy*
[f] *Department of Physics and Materials Science, University of Luxembourg, Belvaux, 4422, Luxembourg*

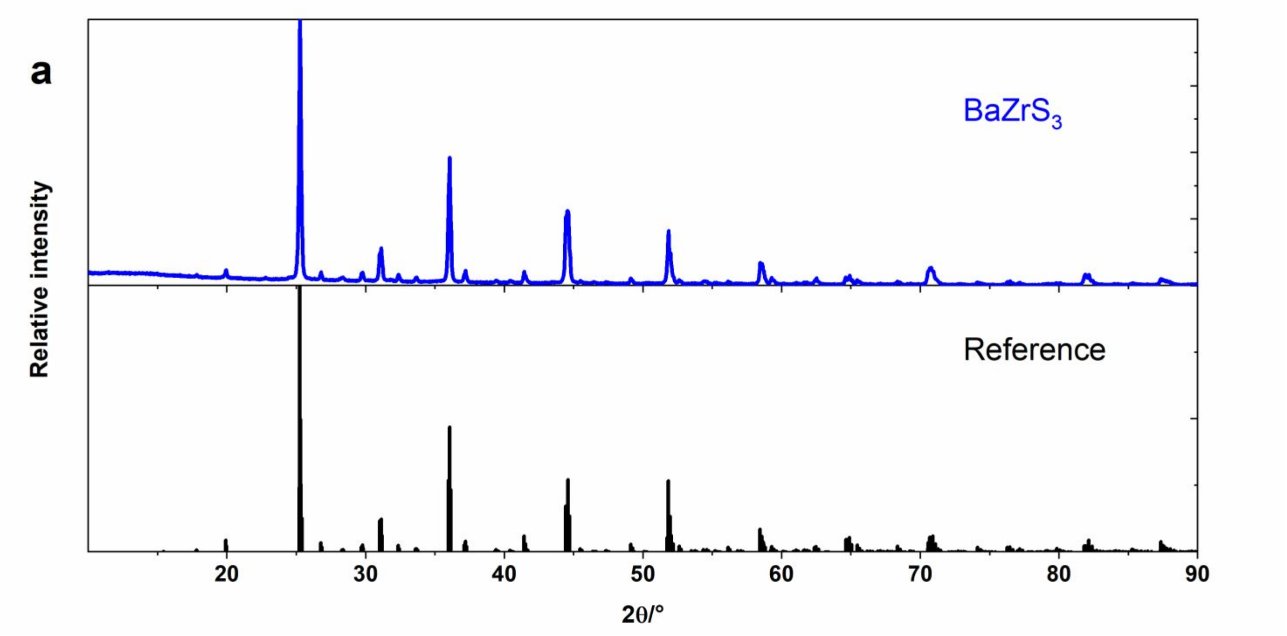

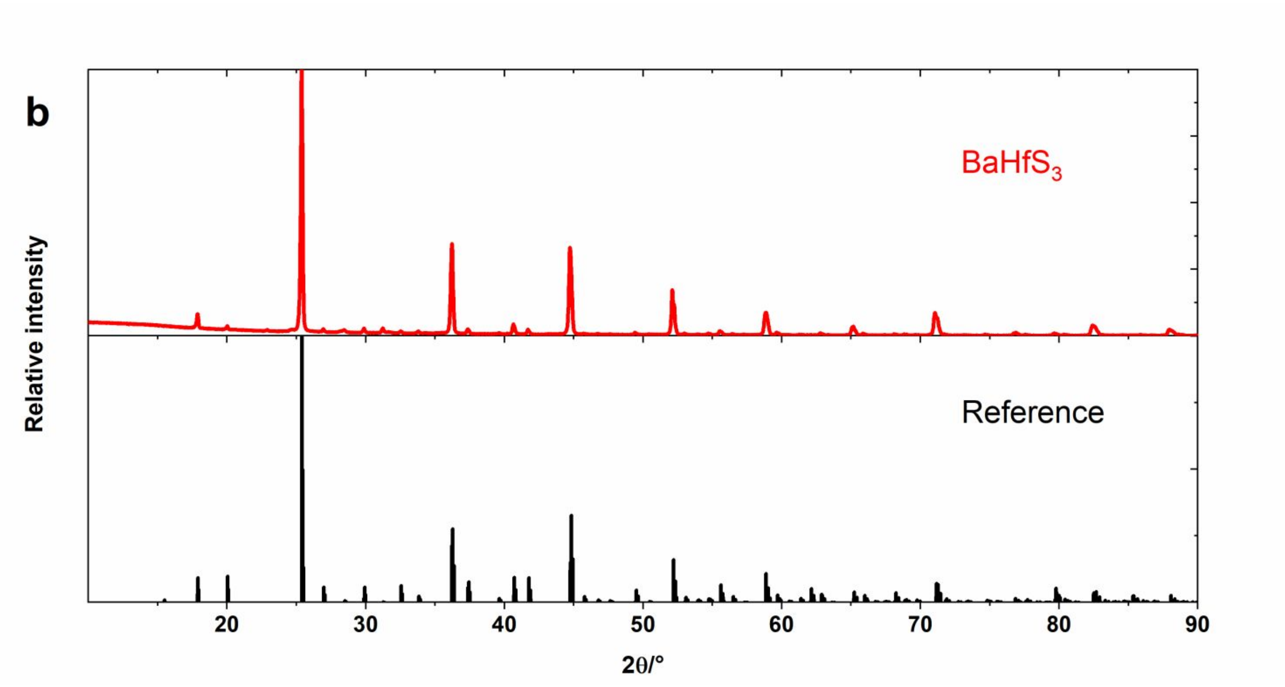

Figure S1. Powder diffraction patterns of as-prepared (a) $BaZrS_3$ and (b) $BaHfS_3$, along with reference patterns.

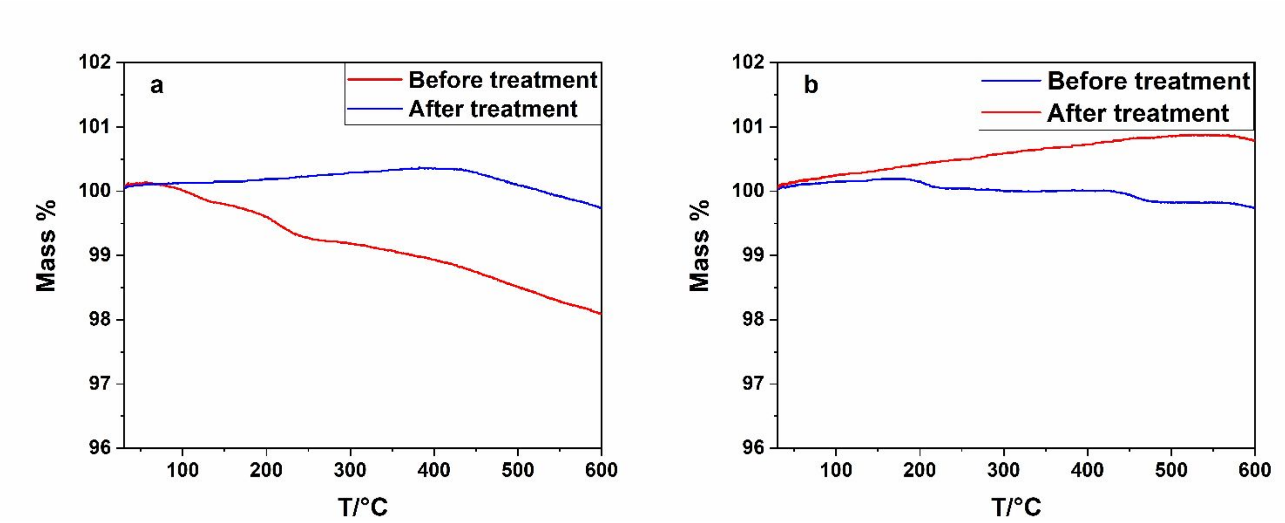

Figure S2. Thermogravimetric (100 $cm^3 \cdot min^{-1}$Ar@STP, 10 $°C \cdot min^{-1}$) curves of (a) $BaZrS_3$ and (b) $BaHfS_3$ before and after heat treatment in high vacuum.

Table S1. Measured molar heat capacity values at constant pressure for $BaHfS_3$. Measurements were performed using a Quantum Design Physical Properties Measurement System (PPMS) with a standard uncertainty of 2% $C_{p,\mathrm{m}}$ below about T = 10 K and 1% $C_{p,\mathrm{m}}$ from T = (10 to 300) K. The standard uncertainty in temperature is about 4 mK.

T/K	$C_{p,\mathrm{m}}$/J·K^{-1}·mol^{-1}	T/K	$C_{p,\mathrm{m}}$/J·K^{-1}·mol^{-1}	T/K	$C_{p,\mathrm{m}}$/J·K^{-1}·mol^{-1}
1.8737	$3.5229 \cdot 10^{-3}$	7.6969	0.40805	75.130	63.129
1.9089	$3.7596 \cdot 10^{-3}$	8.0427	0.48260	82.097	70.090
1.9656	$4.1351 \cdot 10^{-3}$	8.4018	0.58272	89.740	76.650
2.0271	$4.5788 \cdot 10^{-3}$	8.7895	0.70091	98.090	81.591
2.0912	$4.9764 \cdot 10^{-3}$	9.1782	0.82536	107.69	86.449
2.1619	$5.4580 \cdot 10^{-3}$	9.5853	0.98254	115.03	89.420
2.2405	$6.3496 \cdot 10^{-3}$	10.025	1.1803	121.28	90.928
2.3243	$6.8924 \cdot 10^{-3}$	10.500	1.4041	127.86	93.624
2.4129	$7.5927 \cdot 10^{-3}$	10.979	1.6504	134.78	97.082
2.5103	$8.3443 \cdot 10^{-3}$	11.473	1.9288	142.09	99.458
2.6408	$9.3318 \cdot 10^{-3}$	11.986	2.2430	149.81	100.95
2.7806	0.010721	12.516	2.5984	157.94	102.82
2.9144	0.011541	13.072	3.0007	166.51	105.28
3.0467	0.012950	13.657	3.4482	175.54	107.76
3.1826	0.014733	14.265	3.9377	185.05	110.21
3.3235	0.017472	14.898	4.4672	195.06	111.78
3.4695	0.020007	15.452	4.9302	205.65	113.59
3.6220	0.023030	16.587	6.0028	216.80	115.55
3.7839	0.026666	18.134	7.5470	228.55	117.27
3.9566	0.031547	19.817	9.3305	240.94	117.99
4.1373	0.036048	21.659	11.304	254.01	117.88
4.3319	0.043688	23.675	13.482	267.76	119.91
4.5291	0.050240	25.881	15.870	282.24	123.27
4.7400	0.059774	28.284	18.457	297.56	121.65
4.9523	0.071656	30.915	21.576		
5.1804	0.086026	33.791	25.080		
5.4162	0.10219	36.931	28.798		
5.6618	0.12014	40.359	32.960		
5.9185	0.14063	44.117	36.697		
6.1877	0.16957	48.205	40.331		
6.4617	0.19960	52.679	45.032		
6.7577	0.23453	57.573	50.367		
7.0549	0.28269	62.922	54.834		
7.3669	0.34043	68.759	58.429		

Table S2. Measured molar heat capacity values at constant pressure for $BaZrS_3$. Measurements were performed using a Quantum Design Physical Properties Measurement System (PPMS) with a standard uncertainty of 2% $C_{p,m}$ below about T = 10 K and 1% $C_{p,m}$ from T = (10 to 300) K. The standard uncertainty in temperature is about 4 mK.

T/K	$C_{p,m}$/J·K^{-1}·mol^{-1}	T/K	$C_{p,m}$/J·K^{-1}·mol^{-1}	T/K	$C_{p,m}$/J·K^{-1}·mol^{-1}
1.8470	0.017010	7.8636	0.36519	77.358	59.786
1.9140	0.017818	8.2297	0.43863	84.531	66.904
1.9836	0.018135	8.5794	0.51883	92.409	72.923
2.0589	0.019279	8.9632	0.62230	101.00	77.841
2.1379	0.020147	9.3613	0.73247	111.08	83.323
2.2246	0.020907	9.7793	0.87293	117.08	85.502
2.3174	0.021431	10.231	1.0642	123.44	87.704
2.4142	0.023060	10.693	1.2253	130.12	91.115
2.5146	0.023653	11.171	1.4329	137.16	94.687
2.6367	0.024681	11.670	1.6702	144.60	96.998
2.7608	0.025779	12.189	1.9395	152.46	99.049
2.8840	0.026519	12.726	2.2341	160.73	101.67
3.0113	0.029149	13.291	2.5722	169.44	104.46
3.1457	0.028846	13.883	2.9575	178.62	107.26
3.2855	0.030659	14.500	3.3718	188.30	109.82
3.4296	0.033203	15.142	3.8164	198.48	111.64
3.5838	0.035130	15.650	4.1670	209.25	113.77
3.7417	0.036712	17.103	5.2943	220.59	116.23
3.9077	0.041766	18.695	6.6396	232.55	117.95
4.0828	0.042426	20.426	8.1783	245.15	119.16
4.2691	0.047478	22.327	9.8994	258.45	120.28
4.4687	0.054190	24.399	11.779	272.44	122.64
4.6646	0.063304	26.670	13.879	287.16	126.00
4.8715	0.068598	29.140	16.159	302.75	124.50
5.0845	0.076307	31.851	19.017		
5.3093	0.088073	34.809	22.113		
5.5472	0.10407	38.040	25.439		
5.8054	0.11437	41.568	29.294		
6.0651	0.13845	45.441	32.623		
6.3376	0.15518	49.637	36.297		
6.6111	0.18626	54.250	41.099		
6.9016	0.21753	59.286	46.231		
7.2048	0.25394	64.793	50.338		
7.5266	0.30738	70.802	54.403		

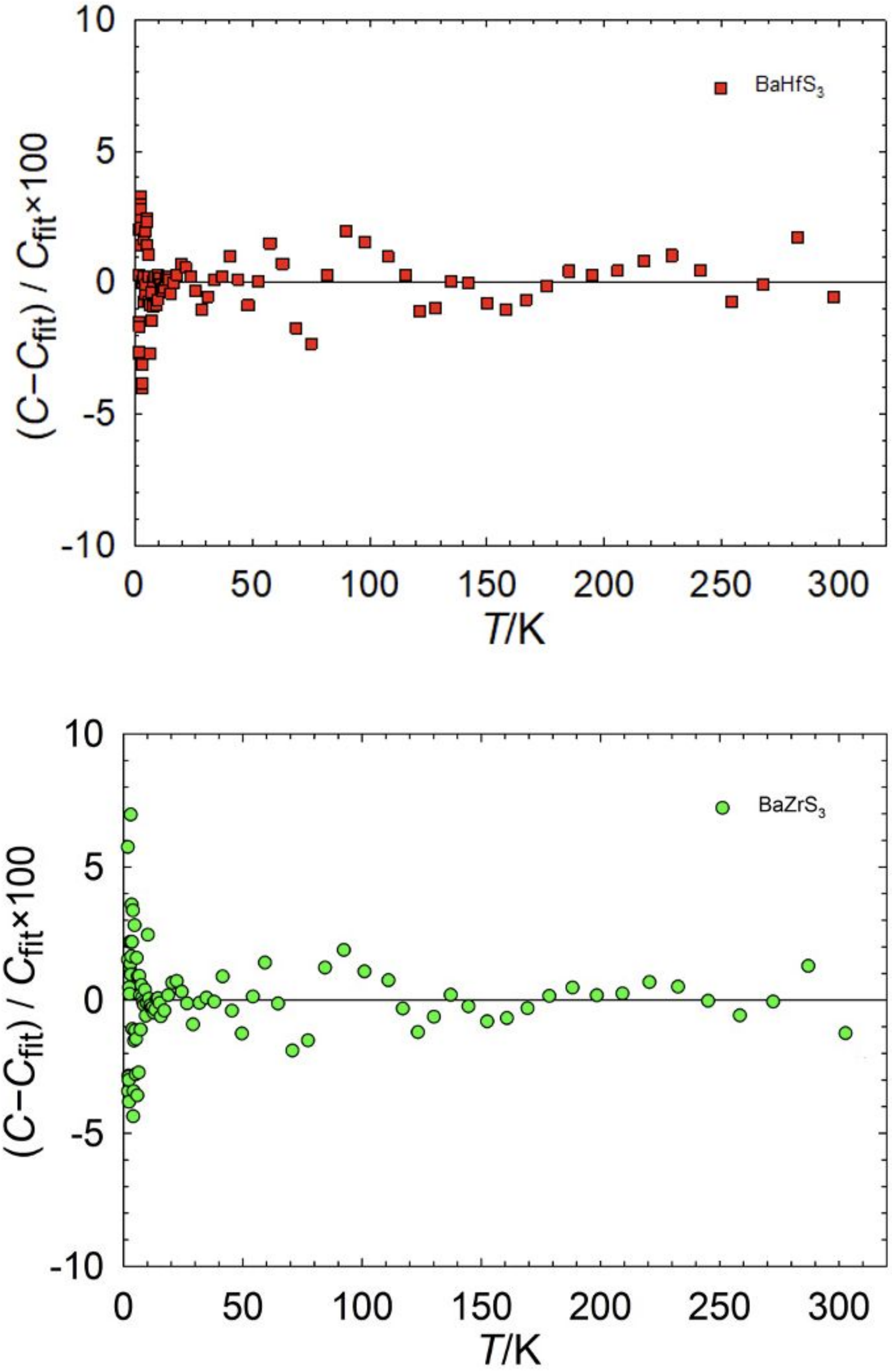

Figure S3. Deviation plots of the heat capacity results from the theoretical fits versus temperature of $BaHfS_3$ and $BaZrS_3$. Parameters for theoretical fits as well as %RMS values are given in Table 2.

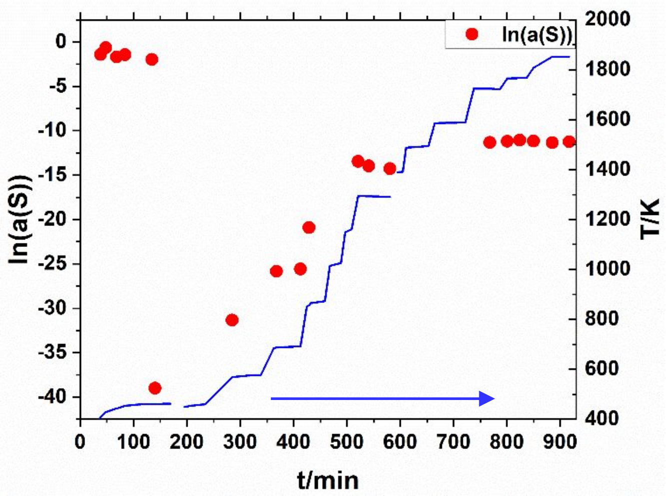

Figure S4. Sulfur activity (expressed as ln(a(S))) as a function of time and temperature in the three experiments carried out on the same $BaZrS_3$ sample.

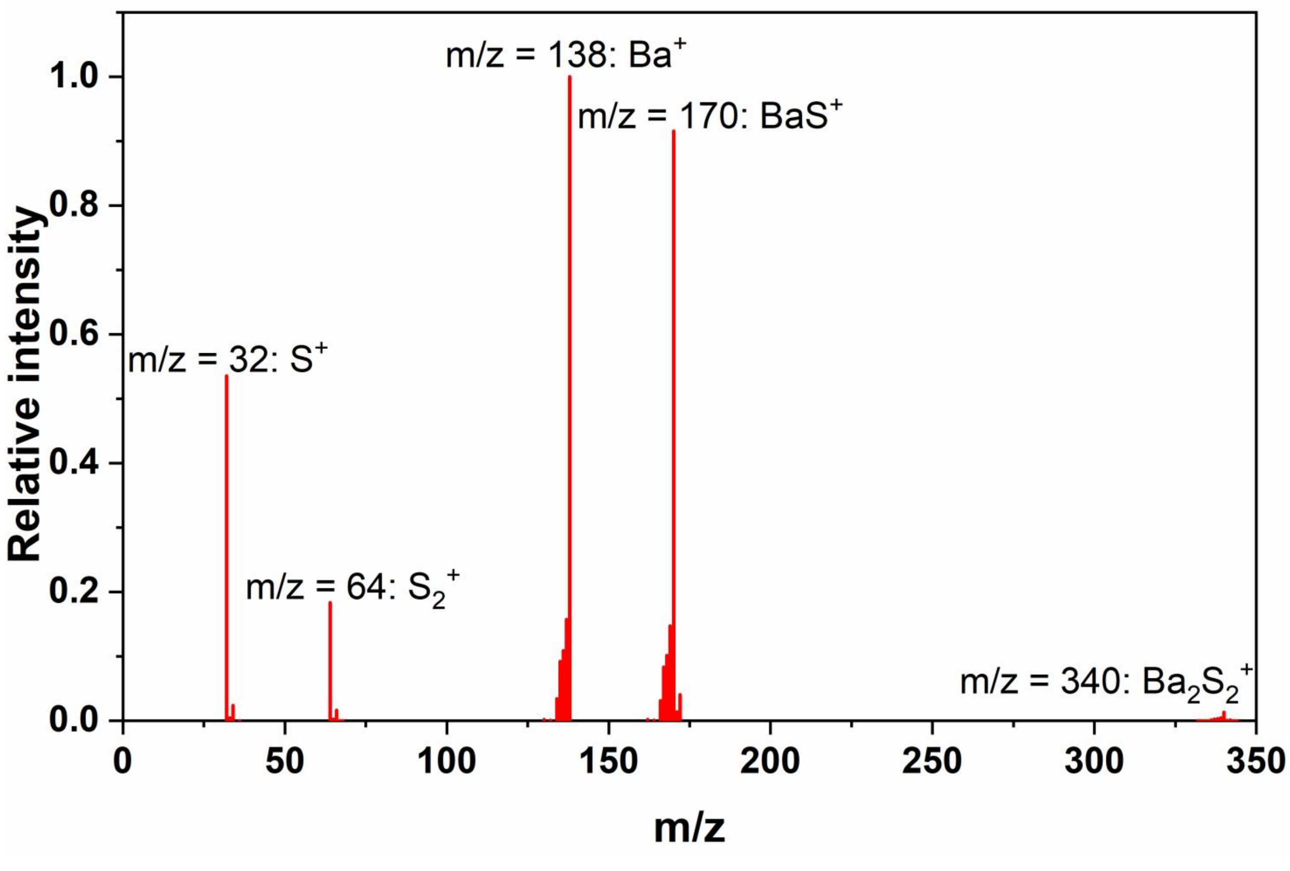

Figure S5. Mass spectrum of the vapor phase above BaS at T = 1910 K.

Table S3. Partial pressures of gaseous species released by $BaZrS_3$ in the first KEMS experiment.

T/K	$P(S_2)$/Pa
409	$4.0 \cdot 10^{-2}$
428	$2.4 \cdot 10^{-1}$
444	$1.6 \cdot 10^{-2}$
444	$2.3 \cdot 10^{-2}$
454	$7.6 \cdot 10^{-2}$
460	$5.9 \cdot 10^{-2}$
460	$6.0 \cdot 10^{-2}$
460	$6.3 \cdot 10^{-2}$
460	$6.4 \cdot 10^{-2}$
461	$6.7 \cdot 10^{-2}$
461	$6.9 \cdot 10^{-2}$
461	$7.0 \cdot 10^{-2}$
461	$6.9 \cdot 10^{-2}$
461	$6.9 \cdot 10^{-2}$
461	$6.7 \cdot 10^{-2}$
462	$4.5 \cdot 10^{-2}$

Table S4. Partial pressures of gaseous species released by $BaZrS_3$ in the second KEMS experiment.

T/K	$P(S_2)$/Pa
449	$2.0 \cdot 10^{-3}$
454	$5.3 \cdot 10^{-3}$
460	$1.4 \cdot 10^{-2}$
569	$3.5 \cdot 10^{-1}$
577	$2.8 \cdot 10^{-2}$
577	$2.7 \cdot 10^{-2}$
685	$4.2 \cdot 10^{-1}$
687	$2.9 \cdot 10^{-1}$
692	$7.6 \cdot 10^{-3}$
851	$9.3 \cdot 10^{-2}$
856	$4.4 \cdot 10^{-2}$
865	$1.2 \cdot 10^{-2}$
1014	$8.1 \cdot 10^{-3}$
1025	$7.5 \cdot 10^{-4}$
1149	$4.6 \cdot 10^{-3}$
1160	$3.6 \cdot 10^{-3}$
1293	$1.0 \cdot 10^{-1}$
1294	$1.0 \cdot 10^{-1}$
1293	$8.0 \cdot 10^{-2}$
1291	$1.4 \cdot 10^{-2}$

Table S5. Partial pressures of gaseous species released by $BaZrS_3$ in the third KEMS experiment.

T/K	P(S)/Pa	$P(S_2)$/Pa	P(Ba)/Pa	P(BaS)/Pa
1389		$7.2 \cdot 10^{-2}$		
1391		$1.2 \cdot 10^{-2}$		
1488		$1.6 \cdot 10^{-1}$		

1489		$7.4\cdot10^{-2}$		
1494		$3.1\cdot10^{-2}$		
1586		$8.5\cdot10^{-2}$		
1588		$4.3\cdot10^{-2}$		
1589		$4.1\cdot10^{-2}$		
1724				
1724				
1724	$3.0\cdot10^{-2}$	$3.1\cdot10^{-1}$		
1722		$2.4\cdot10^{-1}$		
1765	$5.3\cdot10^{-2}$	$6.2\cdot10^{-1}$	$3.5\cdot10^{-4}$	$2.8\cdot10^{-3}$
1766				
1767	$6.2\cdot10^{-2}$	$3.4\cdot10^{-1}$		
1767		$2.5\cdot10^{-1}$		
1808	$8.4\cdot10^{-2}$	$5.2\cdot10^{-1}$	$1.8\cdot10^{-3}$	$5.0\cdot10^{-3}$
1852	$1.1\cdot10^{-1}$	$2.0\cdot10^{-1}$	$5.5\cdot10^{-3}$	$1.0\cdot10^{-2}$
1852	$1.2\cdot10^{-1}$	$1.2\cdot10^{-1}$	$7.2\cdot10^{-3}$	$1.5\cdot10^{-2}$

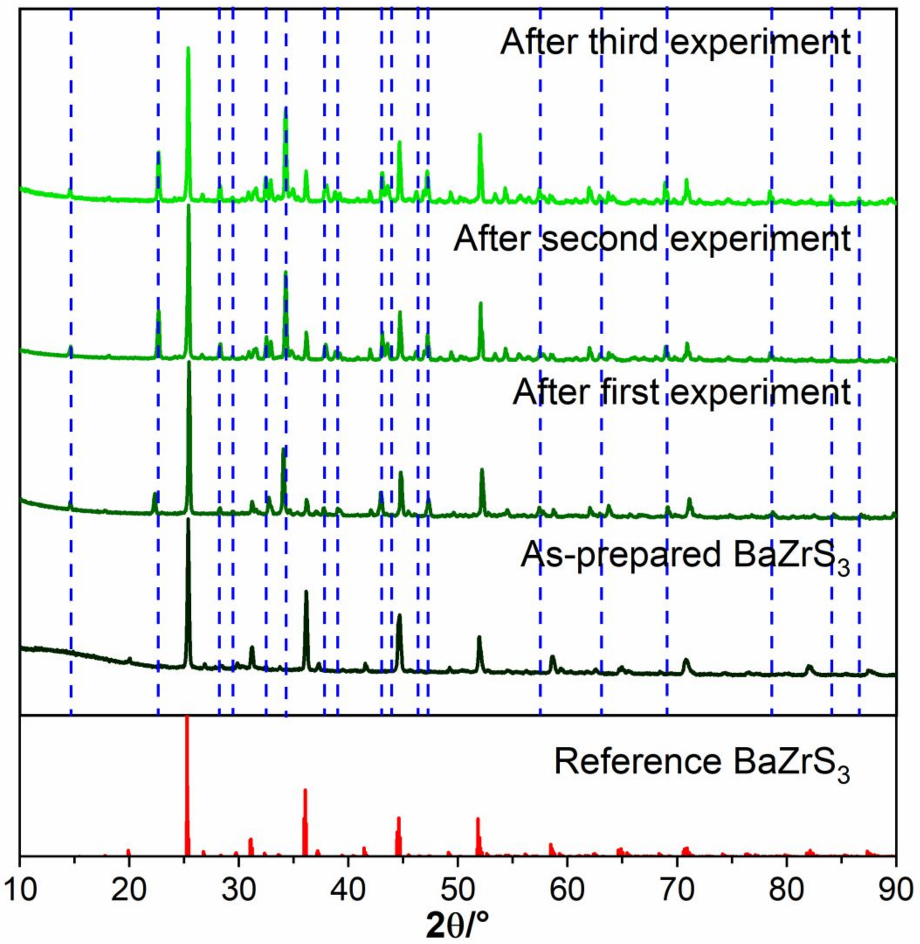

Figure S6. XRD patterns of the as-prepared sample of $BaZrS_3$ and of the same sample after KEMS experiments. New peaks arising from thermal decomposition of $BaZrS_3$ are indicated by the red dashed lines. The reference pattern of $BaZrS_3$ is also shown for comparison.

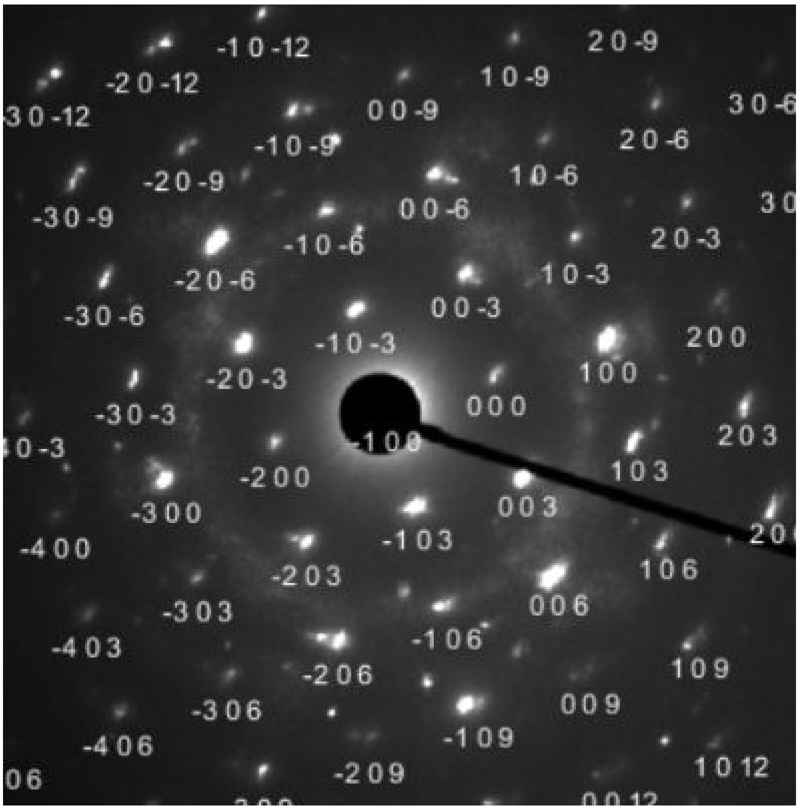

Figure S7. Experimental Ba_2ZrS_4 electron diffraction pattern overlapped to the reference one.

Table S6. Results of SEM-EDX analysis on pristine $BaZrS_3$.

Element number	Element symbol	Element name	Atomic conc.	Weight conc.
16	S	Sulfur	57.1	26.7
40	Zr	Zirconium	18.7	24.8
56	Ba	Barium	24.2	48.5

Table S7. Results of SEM-EDX analysis on solid residue after KEMS experiments on $BaZrS_3$.

Element number	Element symbol	Element name	Atomic conc.	Weight conc.
16	S	Sulfur	46.5	19.2
40	Zr	Zirconium	23.4	27.5
56	Ba	Barium	30.1	53.3
